# Genome-wide association studies for agronomical traits in a world wide spring barley collection

**DOI:** 10.1186/1471-2229-12-16

**Published:** 2012-01-27

**Authors:** Raj K Pasam, Rajiv Sharma, Marcos Malosetti, Fred A van Eeuwijk, Grit Haseneyer, Benjamin Kilian, Andreas Graner

**Affiliations:** 1 Leibniz-Institute of Plant Genetics and Crop Plant Research (IPK), Corrensstr. 3, 06466 Gatersleben, Germany; 2 Biometris, Wageningen UR, P.O.Box 100, Wageningen, The Netherlands; 3 Plant Breeding, Centre of Life and Food Sciences Weihenstephan, Technical University Munich, Freising, Germany

## Abstract

**Background:**

Genome-wide association studies (GWAS) based on linkage disequilibrium (LD) provide a promising tool for the detection and fine mapping of quantitative trait loci (QTL) underlying complex agronomic traits. In this study we explored the genetic basis of variation for the traits heading date, plant height, thousand grain weight, starch content and crude protein content in a diverse collection of 224 spring barleys of worldwide origin. The whole panel was genotyped with a customized oligonucleotide pool assay containing 1536 SNPs using Illumina's GoldenGate technology resulting in 957 successful SNPs covering all chromosomes. The morphological trait "row type" (two-rowed spike vs. six-rowed spike) was used to confirm the high level of selectivity and sensitivity of the approach. This study describes the detection of QTL for the above mentioned agronomic traits by GWAS.

**Results:**

Population structure in the panel was investigated by various methods and six subgroups that are mainly based on their spike morphology and region of origin. We explored the patterns of linkage disequilibrium (LD) among the whole panel for all seven barley chromosomes. Average LD was observed to decay below a critical level (r^2^-value 0.2) within a map distance of 5-10 cM. Phenotypic variation within the panel was reasonably large for all the traits. The heritabilities calculated for each trait over multi-environment experiments ranged between 0.90-0.95. Different statistical models were tested to control spurious LD caused by population structure and to calculate the *P-*value of marker-trait associations. Using a mixed linear model with kinship for controlling spurious LD effects, we found a total of 171 significant marker trait associations, which delineate into 107 QTL regions. Across all traits these can be grouped into 57 novel QTL and 50 QTL that are congruent with previously mapped QTL positions.

**Conclusions:**

Our results demonstrate that the described diverse barley panel can be efficiently used for GWAS of various quantitative traits, provided that population structure is appropriately taken into account. The observed significant marker trait associations provide a refined insight into the genetic architecture of important agronomic traits in barley. However, individual QTL account only for a small portion of phenotypic variation, which may be due to insufficient marker coverage and/or the elimination of rare alleles prior to analysis. The fact that the combined SNP effects fall short of explaining the complete phenotypic variance may support the hypothesis that the expression of a quantitative trait is caused by a large number of very small effects that escape detection. Notwithstanding these limitations, the integration of GWAS with biparental linkage mapping and an ever increasing body of genomic sequence information will facilitate the systematic isolation of agronomically important genes and subsequent analysis of their allelic diversity.

## Background

Determining the genetic basis of agronomic traits has been one of the major scientific challenges in the process of crop improvement . Most of the agronomically important traits are quantitative, resulting in greater difficulty for discerning genetic differences underlying the phenotype of interest. Currently, linkage mapping (analysis) is the most common approach in plants to detect quantitative trait loci (QTL) corresponding to complex traits. In linkage mapping, linkage disequilibrium (LD) is generated by establishing a population from a cross between two parental lines. The co-segregation of alleles of mapped marker loci and phenotypic traits allows the identification of linked markers. Due to the restricted number of meiotic events that are captured in a biparental mapping population, the genetic resolution of QTL maps often remains confined, to a range of 10-30 cM [1,2]. Moreover, linkage analysis can only sample a small fraction of all possible alleles in a population from which the parents originated.

An alternative approach, association mapping (AM) known as LD mapping relies on existing natural populations or designed populations of plants to overcome the constraints inherent to linkage mapping. LD mapping exploits ancestral recombination events that occurred in the population and takes into account all major alleles present in the population to identify significant marker-phenotype associations. LD mapping was first introduced in genetic mapping studies in humans [3,4] and has been recently considered for plant research. By exploiting non-random associations of alleles at nearby loci (LD), it is possible to scoop out significantly associated genomic regions with a set of mapped markers. Success of mapping depends on the quality of phenotypic data, population size and the degree of LD present in a population [5,6]. In general, the power of association studies depends on the degree of LD between genotyped markers and the functional polymorphisms. The decay of LD varies greatly i) between species [7], ii) among different populations within one species and iii) also among different loci within a given genome [8,9].

LD mapping is based on two strategies: i) re-sequencing of selected candidate genes and ii) genome-wide association which exploits marker polymorphisms across all chromosomes [10]. Genome-wide association studies (GWAS) have become increasingly popular and powerful over the last few years in human and animal genetics. The emergence of more cost-effective, high-throughput genotyping platforms have rendered AM an increasingly attractive approach for QTL mapping in plants [11]. In the last few years, an increasing number of association studies based on the analysis of candidate genes have been published (reviewed in [7]). These include e.g. the *Dwarf8 *[12] and the phytoene synthase locus in maize [13], flowering time genes in barley [14], the *PsyI*-*AI *locus in wheat [15], the *rhg-1 *gene in soybean [16]; and a series of candidate genes in *Arabidopsis *[17,18].

Barley (*Hordeum vulgare *L.) was domesticated in the Fertile Crescent about 10,000 years ago [19-21]. Today barley is the fourth most important cereal crop after wheat, rice and maize. In addition to its agricultural importance, the barley genome is considered as a model for other crop species of the Triticeae tribe including wheat and rye [22,23]. In this regard an ever increasing repertoire of marker and sequence resources has been developed for barley which can be efficiently utilized [24-26]. Over the last few years candidate gene based AM studies were reported for barley [9,14,27]. GWAS with dense marker coverage are not yet conducted routinely for barley, albeit the potential of this approach has been demonstrated in some pilot studies [28-30].

Inbreeding crops such as barley are characterized by a high level of population structure caused by the impact of non random mating and subsequent selection. This is exemplified by two-rowed and six-rowed barley cultivars which form distinct subpopulations, because the corresponding breeding programs rely on different progenitors. The same applies to the subpopulations of spring and winter barley [31]. There are higher chances of occurrence of type I and type II errors in AM than in biparental QTL analysis due to the confounding effect of population structure in the panel [2,32-34] Specific statistical approaches have been proposed to account for population structure in AM [35]. Yu et al. [36] described a mixed-linear model (MLM) approach which performs better than previous models [37]. Still these models have their individual shortcomings and care needs to be taken in controlling for population structure and balancing the rate of false positives and false negatives in the analysis.

In the present study, our main objective was to map genetic polymorphisms underlying complex agronomic traits such as heading date (HD), plant height (PHT), thousand grain weight (TGW), starch content (SC) and crude protein content (CPC) in spring barley using GWAS. We studied a diverse spring barley collection comprising 224 accessions from 52 countries previously described by Haseneyer et al. [38]. We provide a comprehensive overview on population structure and genetic diversity as well as their effects on GWAS. To study the dynamics of LD across the seven barley chromosomes we investigated the patterns of LD decay. Finally, we identify and locate a substantial number of known and novel QTL for the traits investigated.

## Methods

### Association mapping panel

The association mapping panel consists of 224 spring barley accessions selected from the Barley Core Collection (BCC) [39] and the barley Genebank collection maintained at the IPK Genebank Gatersleben, Germany. The panel comprises 96 two-rowed and 128 six-rowed genotypes, and among them 109 accessions originate from Europe (EU), 45 from West Asia and North Africa (WANA), 40 from East Asia (EA) and 30 from the Americas (AM). Most of the accessions are improved cultivars (149), some accessions are landraces (57) or breeder's lines (18). Further information on the germplasm can be obtained from the European Barley Database (EBDB, http://barley.ipk-gatersleben.de/ebdb.php3). This panel has been considered and described in detail by Haseneyer et al. [38]. Each accession has been single-seed descended, selfed for two generations under greenhouse conditions and subsequently propagated in the field.

### Phenotypic evaluation

The accessions were planted in a 25 × 15 lattice design with three replications in the years 2004 and 2005 at the following locations: Stuttgart (Southwest Germany), Irlbach (Southeast Germany) and Wohlde (Northern Germany). Heading date (HD) and plant height (PHT) were scored in field plots. Thousand grain weight (TGW) was estimated from sampled grains per plot. Starch content (SC) and crude protein content (CPC) were estimated using a near infrared reflectance spectrometer (NIRS) from ground seed samples from all environments. In order to convert the nitrogen content to crude protein values, we considered a factor of 6.25. We followed the methods described in Naumann and Bassler [40] to estimate the starch content and nitrogen content. Phenotypic data were analyzed using REML (Residual Maximum Likelihood) implemented in GenStat 9 software [41]. Variance components were calculated by fitting a mixed linear model (MLM) to multi-environment data. Heritabilities were estimated for all traits considering the percentages of genotypic variance, over the total phenotypic variance including genotype (G) by environment (E) variance and error variance components. Phenotypic mean BLUEs (Best Linear Unbiased Estimates) were estimated taking into account the GxE variance and were used for association studies. Further information on phenotypic data can be obtained from Haseneyer et al. [38].

### Genome-wide marker profiling

DNA for SNP genotyping was extracted for each accession from bulked leaf samples of eight 2-weeks old seedlings. A customized oligonucleotide pool assay (IPK-OPA, unpubl) containing 1536 allele specific oligos was used to genotype the panel by Illumina's GoldenGate technology (Illumina, San Diego, CA). The IPK-OPA has been mainly built on a selection of markers from two pilot assays (pOPA1, pOPA2) that are polymorphic between the two barley cultivars 'Barke' and, 'Morex'. More than 95% of the 1536 SNP markers of the IPK-OPA have been included in a barley consensus map [26]. The SNP genotyping was performed at University of California (Southern California Genotyping Consortium, UCLA) following the protocol of Fan et al. [42,43]. More details about the successful SNP markers considered for GWAS are available as supplemental information (Additional file 1: Table S1).

Scoring SNP data was done using the Illumina Beadstudio package (Genotyping module 3.2.32; Genome viewer 3.2.9; Illumina, San Diego, CA) that can process the raw hybridization intensity data and thereby cluster the data. The normalization procedure implemented in the Beadstudio genotyping module includes outlier removal, background correction and scaling. The algorithm included uses a Bayesian model to assign normalized intensity values to one of the three possible homozygous and heterozygous genotype clusters. Stringent threshold scores (*Call Rate *> 0.9 and *GenTrain Score *> 0.7) were used to identify ambiguous results. SNPs that failed to show two-group clustering were strictly excluded from the analysis. From a total of 1536 SNP markers, 985 markers yielded good quality genotypic calls. Among the 985 successful SNP markers only 957 markers are genetically mapped and we used these 957 markers for our analysis (Additional file 1: Table S1). Among the 224 accessions in the panel of genotypes, 12 genotypes performed badly in the assay (Additional file 2: Table S2). For these 12 genotypes more than 90% of the SNP markers data is missing, hence were excluded from subsequent analysis.

### Genotypic data analysis and population structure

Polymorphic information content (PIC) values were calculated for each SNP using Powermarker 3.25. [44]. Major allele frequency, minor allele frequency (MAF), gene diversity and Nei's genetic distance (*d*) [45] were calculated and a NJ (Neighbor-Joining) dendrogram (data not shown) based on *d *was computed. From the 957 SNPs, a final set comprising 918 SNPs with MAF larger than 0.05 was used for analysis of population structure, LD and marker trait associations.

To estimate the number of subgroups in the panel, different methodologies and different software packages were employed and compared in order to determine the appropriate population structure in collection. For the quantitative assessment of the number of groups in the panel, a Bayesian clustering analysis was performed using a model based approach implemented in the software package STRUCTUREv 2.2 [46,47]. This approach uses multi-locus genotypic data to assign individuals to clusters or groups (*k*) without prior knowledge of their population affinities and assumes loci in Hardy-Weinberg equilibrium. The program was run with 918 SNP markers for *k*-values 1 to 15 (hypothetical number of subgroups), with 100000 burnin iterations followed by 50000 MCMC (Markov Chain Monte Carlo) iterations for accurate parameter estimates. To verify the consistency of the results we performed 5 independent runs for each *k*. An admixture model with correlated allele frequencies was used. The most probable number of groups was determined by plotting the estimated likelihood values [LnP(D)] obtained from STRUCTURE runs against *k*. LnP(D) is the log likelihood of the observed genotype distribution in *k *clusters and is an output by STRUCTURE simulation. The *k *value best describes the population structure based on the criteria of maximizing the log probability of data or in other words the value at which LnP(D) reaches a plateau [46]. STRUCTURE results with the SNP marker dataset were confirmed with the results from STRUCTURE runs using a set of Diversity Array Technology (DArT) markers (Pasam et al. unpubl, Additional file 3: Figure S1). In a second approach principal coordinate analysis (PCoA) based on the dissimilarity matrix was performed using DARwin (Diversity Analysis and Representation for windows) [48]. In a third approach a NJ dendrogram based on Nei's genetic distance matrix was constructed. The substructure in the collection using different methodologies was compared and the final *k *value using STRUCTURE was ascertained. For this *k *value, the Q-matrix (population membership estimates) was extracted from STRUCTURE runs. This matrix provides the estimated membership coefficients for each accession in each of the subgroups.

### Linkage disequilibrium analysis

The extent of LD effects both the number of markers required for GWAS and the resolution of mapping the trait. LD is in many cases influenced by population structure resulting from the demographic and breeding history of the accessions. Genome-wide LD analysis was performed among the panel and subgroups by pair wise comparisons among the SNP markers using HAPLOVIEW [49]. LD was estimated by using squared allele frequency correlations (*r*^2^) between the pairs of loci [50]. The loci were considered to be in significant LD when *P *< 0.001, the rest of *r*^2 ^values were not considered as informative. The pattern and distribution of intra-chromosomal LD was visualized and studied from LD plots generated for each chromosome by HAPLOVIEW. To investigate the average LD decay in the whole genome among the panel, significant intra-chromosomal *r*^2 ^values were plotted against the genetic distance (cM) between markers. The smothering second degree LOESS curve was fitted using GENSTAT [41]. A critical value for *r*^2 ^was estimated by square root transforming of unlinked *r*^2 ^values to obtain a normally distributed random variable, and the parametric 95th percentile of that distribution was taken as a critical *r*^2 ^value [32]. Unlinked *r*^2 ^refers to the *r*^2 ^between the marker loci with a genetic distance greater than 50 cM or on independent linkage groups.

### Association analysis

Different statistical models were used to calculate *P-*values for associating each marker with the trait of interest, along with accounting for population structure to avoid spurious associations by TASSEL v.2.1 (http://www.maizegenetics.net). We followed the formula *y = Xβ+M + Zu + e*, where *y *is a response vector for phenotypic values, *β *is a vector of fixed effects regarding population structure, α is the vector of fixed effect for marker effects, *u *is the vector of random effects for co-ancestry and *e *is the vector of residuals. *X *can be either the Q-matrix or the PCs from Principal Component Analysis (PCA), *M *denotes the genotypes at the marker and *Z *is an identity matrix. Six models comprising both general linear models (GLM) and mixed linear models (MLM) were selected to test the marker-trait-associations (MTA). Results were compared to determine the best model for our analysis. PCA was conducted with TASSEL. The first ten significant PCs explained 43% of the cumulative variance of all markers. A kinship matrix (K-matrix), the pair-wise relationship matrix which is further used for population correction in the association models was calculated with 918 SNP markers using TASSEL [51]. The following models were tested: i) Naive model: GLM without any correction for population structure; ii) Q-model: GLM with Q-matrix as correction for population structure; iii) P-model: GLM with PCs as correction for population structure; iv) QK-model: MLM with Q-matrix and K-matrix as correction for population structure; v) PK-model: MLM with PCs and K-matrix as correction for population structure and vi) K-model: MLM with K-matrix as correction for population structure [35,36,52,53]. All SNP markers were re-mapped by association mapping to determine the mapping resolution of the panel as suggested by [24]. The critical *P-*values for assessing the significance of MTAs were calculated based on a false discovery rate (FDR) separately for each trait [54], which was found to be highly stringent. Considering the stringency of the model used for accounting for population structure, most of the false positives were inherently controlled. Thus, we considered a more liberal approach as proposed by Chan et al. [55] for determining the threshold level for significant MTAs. It was suggested that the bottom 0.1 percentile distribution of the *P-*values can be considered as significant, which in our analysis resulted in threshold levels of 0.05 to 0.09 for individual traits. Alternatively, as a compromise between the two approaches an arbitrary threshold *P-*value of 0.03 was used for all traits and all models. This rather rough estimate was obtained by arranging-log10 *P-*values in a descending order, and the value at which the curve starts to flatten is determined as the threshold value. All association models with all traits were re-analyzed using GENSTAT [41] to check for any discrepancy.

## Results

### Phenotypic data

Large phenotypic variation was observed for all traits. Outliers in the data were identified based on the residuals derived from the data of all environments and were removed from further analysis. For the trait heading date, data from year 2004 was excluded from the analysis due to differences in scoring this trait between the individual locations. Variance components were calculated by REML. The results confirmed that genotypic variance was significant for all traits (*P *< 0.001). GxE interactions were also significant (*P *< 0.001) but represented only a small fraction of the total variance. Heritabilities ranged between 0.90-0.95 indicating the robustness of the data and the low error rate. Year-wise means, ranges and heritabilities over all environments for the traits HD, PHT, TGW, SC and CPC are presented in Table 1 and their frequency distributions are illustrated in Additional file 4: Figure S2. The correlation exhibited by the agronomic traits between each other is outlined in Table 2. The traits SC and CPC are highly correlated (-0.7) and other traits showed moderate to weak correlation among each other. PHT was shown to be weakly correlated with HD and also with SC and CPC. TGW is found to be positively correlated with SC and negatively correlated with CPC. Substantial phenotypic differences were reported between two-rowed and six-rowed genotypes. The means for all traits were significantly different between the two groups (Additional file 5: Table S3). The variation observed was larger for all traits in six-rowed barleys than in two-rowed barleys. The greatest influence of spike morphology (two-rowed vs. six-rowed) on phenotypic variation was seen for TGW, whereas the greatest influence of population structure was observed for PHT (Additional file 6: Table S4).

**Table 1:** Estimation of mean, minimum (Min), maximum (Max) and heritabilities (h^2^) of all traits

Trait	2004 Min	Max	Mean	2005 Min	Max	Mean	h^2^(%) GxE
Plant height (PHT)	20	120	77.04	30	120	73.69	92.82
Heading date (HD)	*	*	*	56	81	68.26	92.5
Thousand grain weight (TGW)	17.77	67.23	44.92	20.1	62.6	42.43	92.9
Starch content (SC)	40.8	64.58	56.85	44.01	65.31	56.91	96.3
Protein content (CPC)	9.74	25.74	14.88	10.35	25.18	14.90	92.1

Heritabilities were calculated on entry mean basis.

**Table 2:** Correlation coefficients among different traits estimated across all environmental data

	HD	PHT	CPC	SC
HD				
PHT	0.29**			
CPC	-0.43**	-0.25**		
SC	0.43**	0.17*	-0.76**	
TGW	0.04	-0.09	-0.30**	0.33**

**highly significant at *P *< 0.001, * significant at *P *< 0.01, rest are not significant

Best Linear Unbiased Estimates (BLUEs) of genotypic means were calculated from the fixed genotypic effects to avail unbiased mean estimates. Using Best Linear Unbiased Predictors (BLUPs) is less suitable as it would cause double shrinking [56]. Henceforth we used BLUEs in our further analysis. However, comparison of both BLUPs and BLUEs revealed very high concordance between both estimates, which is a direct consequence of the high heritabilities (Additional file 7: Figure S3).

### Population structure and genetic diversity

**Figure 1:**
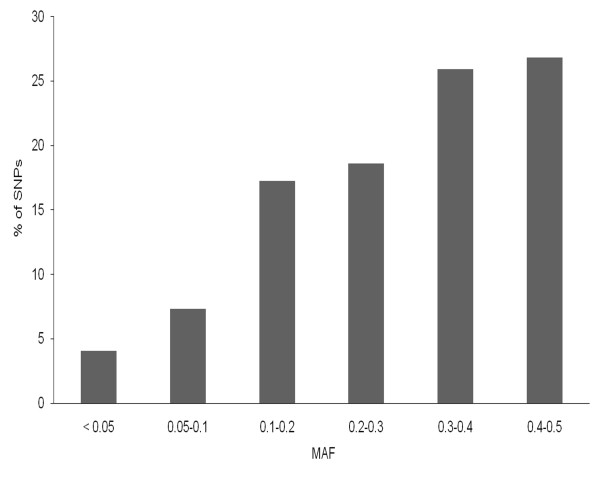
**SNP marker efficiency in the panel. Percentage of distribution of MAF of SNPs in the panel**. SNPs with MAF < 0.05 were excluded from the analysis

From the high quality 985 SNPs, 957 markers had been genetically mapped and therefore were considered for this study. Of these, 39 SNPs (4%) were excluded because of a MAF below 0.05. Of the remaining SNPs, the majority revealed a MAF between 0.1 to 0.5 (Figure 1). These SNP markers were distributed over all seven chromosomes with an average spacing of 1.18 cM. The distribution of SNP markers is not exactly uniform and varies within and among chromosomes with a minimum of 105 markers on chromosome 4H and a maximum of 164 markers on 5H (Table 3). Diversity statistics computed for each SNP are summarized in Additional file 8: Table S5. PIC values for SNPs ranged from 0.09 to 0.5 with an average of 0.30. Most of the markers (726) displayed PIC values exceeding 0.25, demonstrating the informativeness of these markers in our panel. The average PIC values of the markers on each chromosome ranged between 0.29 (5H) to 0.33 (6H). The mean gene diversity value for the whole panel was 0.39 and spread within a range of 0.09 to 0.5. It was reported in several studies that the stratification of barley cultivars is concordant with spike morphology, mainly as a result of breeding history [57,58]. Therefore, similar molecular diversity statistics were generated separately for two-rowed and six-rowed barley groups within our panel and for the six subgroups. Observed mean PIC values are higher for the six-rowed group (0.31) than for two-rowed barleys (0.27). Similarly, average gene diversity estimated was higher in six-rowed (0.38) than in two-rowed accessions (0.33).

**Table 3:** SNP coverage and distribution across all chromosomes

Chromosome	cM	Markers	Marker coverage	PIC
1H	139.79	117	1.19	0.31
2H	156.72	146	1.07	0.29
3H	173.17	151	1.15	0.32
4H	123.29	105	1.17	0.32
5H	195.42	164	1.19	0.29
6H	129.38	119	1.09	0.33
7H	166.56	116	1.44	0.30
Total	1084.33	918	1.18	0.31

Average PIC values for all SNPs on each chromosome are represented.

**Figure 2:**
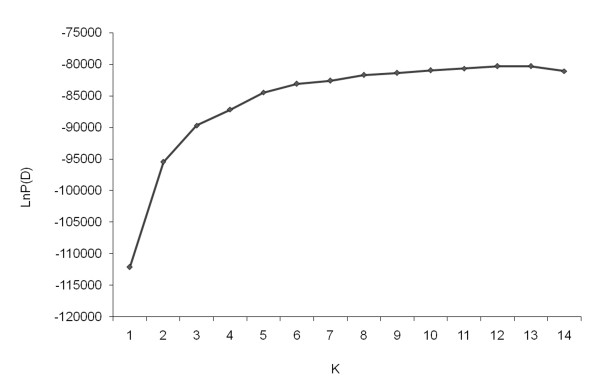
**STRUCTURE results using 918 SNPs**. Log probability data (LnP(D)) as function of *k *(number of clusters) from the STRUCTURE run. The plateau of the graph at *K *= 6 indicates the minimum number of subgroups possible in the panel

**Figure 3:**
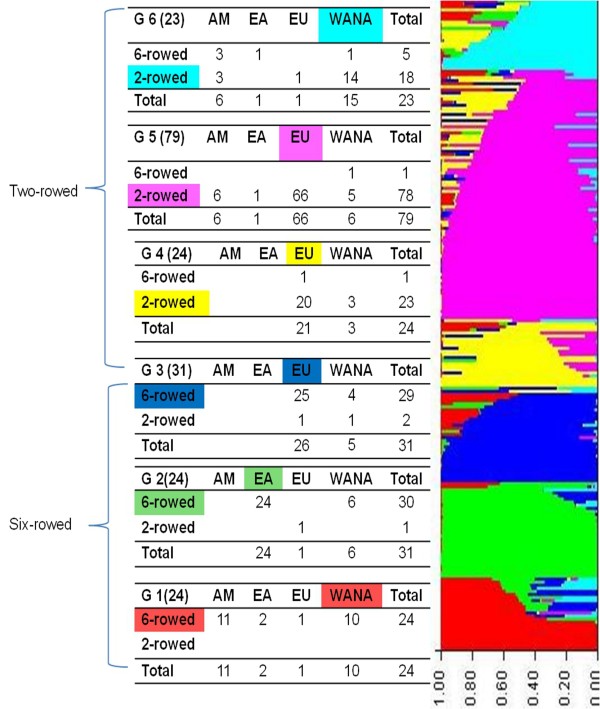
**Population sub-structuring in the panel. **Bayesian clustering of the 212 barley accessions into six defined groups (G1, G2, G3, G4, G5, G6) based on 918 SNP markers. The number of accessions per group and their respective geographical origin and row type is presented

The population structure in the panel of 212 barley accessions was analyzed using 918 SNP markers and a model based approach in STRUCTURE. The LnP(D) appeared to be an increasing function of *k *for all the values observed. But the most significant increase of LnP(D) was observed when *k *was increased from 1 to 2 (Figure 2). At *k *= *= *2 the panel is clearly categorized into two-rowed and six-rowed barleys with few exceptions. The two main groups were further divided yielding six subgroups in total as LnP(D) values nearly reached a plateau at *k *= 6. Hence, we chose a value of *k *= 6 for our analysis as minimum number of groups present in the panel. Different values of *k *are still possible but will not qualitatively affect the results. An accession was assigned to a subgroup if at least 50% of the genome information was estimated to belong to one group. The accessions clustered into groups mostly according to their spike morphology and their geographical origins, as was demonstrated already by Haseneyer et al. [38]. The six groups are defined as: Group 1 (G1): 24 six-rowed barleys mostly from AM and WANA; G2: 31 accessions mostly six-rowed barley from EA; G3: 31 accessions mostly six-rowed barleys from EU; G4: 24 accessions mostly two-rowed from EU; G5: 79 accessions mostly two-rowed barleys from EU; G6: 23 accessions mostly two-rowed from WANA and AM (Figure 3). The dominant stratification of the population according to spike morphology is confirmed by PCoA (Additional file 9: Figure S4) and NJ dendrogram (not shown). In the PCoA, it is obvious that the primary axis separates the accessions based on row type and further grouping is related to the region of origin. Overall, the clustering of accessions was consistent among various methods and we further explored the genetic diversity within these groups. The summary statistics for each group with 918 SNP markers is reported in Table 4. Observed gene diversity values ranged from 0.27 in G5 to 0.35 in G1; PIC values ranged from 0.22 in G5 to 0.29 in G1. Pairwise genetic distances ranged from 0.006 to 0.628, with an overall mean of 0.39. The average overall genetic distance between groups has been calculated, and the largest genetic distance of 0.36 was observed between the groups G2 (six-rowed, EA) and G5 (Two-rowed, EU). Similarly G4 (six-rowed, EU) and G5 (six-rowed, EU) are found to be closely related groups with an average genetic distance of 0.17 (Table 5).

**Table 4:** Summary of molecular diversity and polymorphism information for the whole panel and all the subgroups

Group	Average major allele frequency	No. genotypes	Gene diversity	PIC
Whole panel	0.6978	212	0.3904	0.3079
2-rowed group	0.7551	122	0.3325	0.2714
6-rowed group	0.7064	90	0.3852	0.3108
G1	0.7359	24	0.3551	0.2903
G2	0.7933	31	0.2844	0.2338
G3	0.7773	31	0.3060	0.2497
G4	0.7746	24	0.3106	0.2536
G5	0.7976	79	0.2791	0.2297
G6	0.7547	23	0.3296	0.2681

PIC values are given as the mean values of the corresponding marker partitions.

**Table 5:** Estimation of average genetic distance between different groups

Group	G 1	G 2	G 3	G 4	G 5
G 2	0.24				
G 3	0.24	0.30			
G 4	0.27	0.30	0.29		
G 5	0.31	0.36	0.35	0.17	
G 6	0.21	0.24	0.27	0.21	0.26

### Linkage disequilibrium

**Figure 4:**
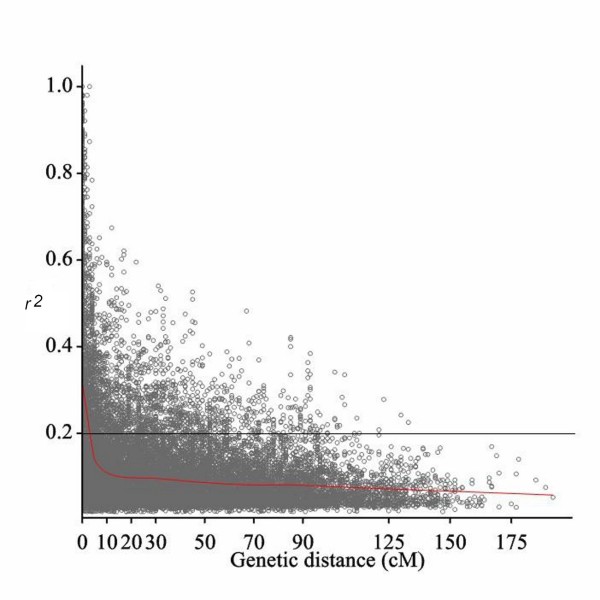
**Intra-chromosomal LD (r**^**2**^**) decay of marker pairs over all chromosomes as a function of genetic distance (cM)**. The horizontal line indicates the 95th percentile distribution of unlinked r^2^. The LOESS fitting curve (red line) illustrates the LD decay

LD analysis was performed using 918 SNPs for i) entire panel, ii) separately for two-rowed and six-rowed barleys, and iii) each of the six subgroups. Pairwise LD was estimated using the squared-allele frequency correlations (r^2^) and was found to decay rapidly with the genetic distance. We studied different aspects of LD in our panel and observed that LD varies along the chromosomes with regions of high LD interspersed with regions of low LD (Additional file 10: Figure S5). A critical value of *r*^2^, or basal LD, was calculated from inter-chromosomal LD analysis and is estimated to be 0.2 beyond which LD is assumed to be caused by genetic linkage. The point at which the LOESS curve intercepts the critical *r*^2 ^is determined as the average LD decay of the population. Based on these criteria the intra-chromosomal LD decayed between 5- 10 cM for individual chromosomes and average LD decay of the whole genome was observed to be at 7 cM (Figure 4). Extensive variability in the magnitude of *r*^2 ^at a given genetic distance was detected reflecting the wide local variation in the extent of LD across the chromosomes. The correlation between *r*^2 ^and marker distance was found to be significantly negative (*r *= -0.40) for markers below a distance of 10 cM, whereas marker pairs with larger distance showed no significant correlation with *r*^2^.

Significant intra-chromosomal *r*^2 ^values (*P *< 0.001) ranged from 0.02 to 1 with an average of 0.12 for the whole panel. Among all significant loci in LD, 13.7% of the loci are above the critical *r*^2 ^value of 0.2 in the whole panel. Pairs of loci are classified into 4 groups based on the inter-marker genetic distance: 0-10 cM (tightly linked markers), 11-20 cM (moderately linked markers), 21-50 (loosely linked markers) and > 50 (independent markers) [59]. The percentages of significant loci pairs and mean *r*^2 ^values for all classes of markers in the whole panel and different subgroups are presented in Table 6. Among all loci pairs, only 39.4% were in significant LD in the whole panel. The percentage of significant loci pairs decreased with the distance between loci; 62.2% of the tightly linked markers showed significant *r*^2^. Similarly 45.1%, of the moderately linked markers 38.3% of the loosely linked markers and 28.5% of independent markers were in significant LD. The portion of *r*^2 ^values exceeding the basal LD level of 0.2 decreased from 33.7% in the group of tightly linked markers to 10% for moderately linked markers to less than 4% for independent markers. Mean *r*^2 ^values decreased from 0.2 for closely linked marker loci to 0.08 for unlinked marker pairs. All loci pairs being in complete LD are spaced at genetic distance < 5 cM.

**Table 6:** LD overview for the whole panel and the subgroups of two-rowed and six-rowed barley

		Total pairs	% significant	Significant pairs	Mean r^2^	Pairs in complete LD	% of pairs in LD > 0.2	Mean of r^2^> 0.2
Whole panel	total	62222	39.4	24567	0.12	59	13.72	0.36
	0-10 cM	10602	62.2	6554	0.20	59	33.70	0.42
	11-20cM	8028	45.1	3626	0.10	0	10.21	0.29
	21-50 cM	19066	38.3	7310	0.09	0	8.40	0.27
	> 50 cM	24526	28.5	7077	0.08	0	4.00	0.25

2-rowed	total	48803	21.6	10544	0.18	94	23.74	0.43
	0-10 cM	8183	50.0	4098	0.29	94	48.00	0.48
	11-20cM	6244	29.9	1869	0.13	0	13.31	0.30
	21-50 cM	15066	17.2	2601	0.12	0	9.59	0.28
	> 50 cM	19310	10.2	1976	0.10	0	3.81	0.26

6-rowed	total	58356	20.2	11801	0.17	95	22.40	0.36
	0-10 cM	9947	36.8	3661	0.24	95	40.37	0.43
	11-20cM	7439	21.1	1569	0.14	0	18.26	0.27
	21-50 cM	17768	16.9	3016	0.14	0	15.04	0.27
	> 50 cM	23202	15.3	3555	0.13	0	12.14	0.25

LD statistics are given for the total number of locus pairs and for different marker linkage classes (for details see text).

### Patterns of linkage disequilibrium within subgroups

At the intra-chromosomal level, mean *r*^2 ^values for two-rowed and six-rowed barley groups ranged between 0.18 and 0.17, which is slightly more than the mean *r*^2 ^of whole panel. The percentages of significant *r*^2 ^values were higher in the two-rowed than in the six-rowed subgroup for all classes of marker pairs except for the independent markers. This pattern is also similar to LD values above the basal level of 0.2, and a slightly slower LD decay was observed for two-rowed barley compared to the group of six-rowed types and to the whole panel. Similarly, the mean *r*^2 ^values were estimated for individual subgroups where they ranged from 0.3 (G5) to 0.49 (G4).

**Figure 5:**
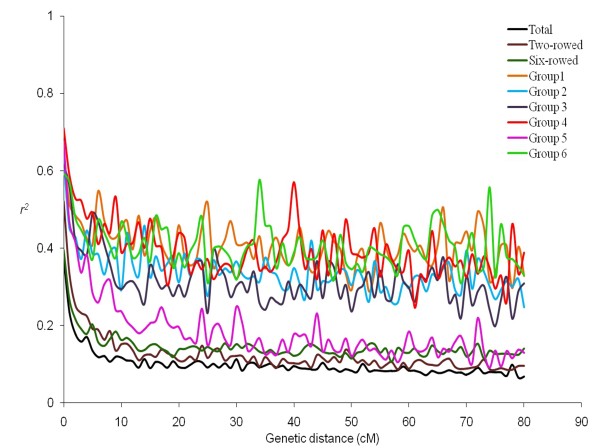
**Comparison of LD patterns and LD decay in the whole panel and subgroups**. Mean r^2 ^values are plotted against the genetic distance for different groups

The LD decay in the subgroups was much slower than in the whole panel. In Figure 5, binned *r*^2 ^values are mapped against the recombination distance (cM) across the genome. In the whole panel the average LD decays below a basal level (0.2) within 5 cM, while in the two-rowed and six-rowed groups the basal level is reached between 10-15 cM and with LD in six-rowed barley decaying faster than in two-rowed barley. Within G5 LD decays to the basal level within 20-25 cM, while it does not reach the basal level in the remaining subgroups (G1,2,3,4,6). Average LD decay graphs for each group showed different patterns. Specifically, in the subgroups G4 and G5 at distances 45 and 74 cM we observed larger LD peaks. Scrutinizing these peaks revealed that high LD in these regions was caused by markers with low allele frequencies. The consequence of the reduced population size of the individual subgroups is that the presence of a solitary allele in single accession already might show a MAF above the critical threshold. Varying patterns of LD decay in different sub-populations are likely reflecting their breeding histories [1] and may impinge on the QTL mapping resolution of the panel.

### Evaluation of the association panel

**Figure 6:**
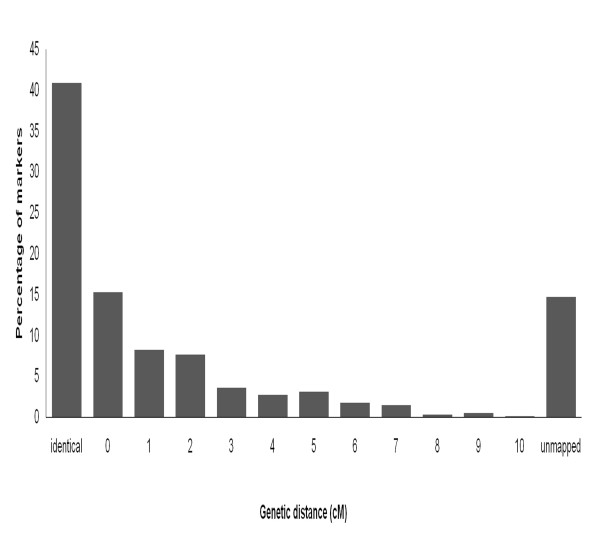
**Evaluating the mapping resolution of the panel**. Distribution of SNPs according to their re-mapped distances using the K-model of genome-wide association approach. The group 'identical' refers to the SNPs that mapped at exactly the same position and the group '0' refers to the SNPs that mapped within a distance of 0.01 to 0.99 cM

All 918 SNPs were re-mapped using an LD approach. A model with kinship accounting for population structure was used for validating the genetic map position of the markers. We used each marker information as an individual trait and ran the analysis with the remaining SNPs to find the most significantly associated markers. The map distance between the target marker in question and the most highly associated marker was used to evaluate the resolution of the panel. More than 85% of the SNP markers had their genetic map position within 0-10 cM distance of their original map position and the majority of them re-mapped at the same position (Figure 6). This re-mapping of markers shows that the resolution of QTL captured by AM approach in our panel will be within a range of 5-10 cM.

### Association analysis

#### Comparison of models

**Figure 7:**
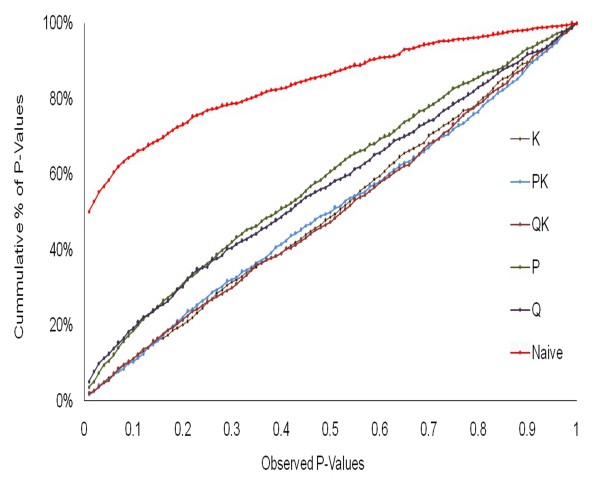
**Comparison of different GWA models**. Cumulative distribution of P-values computed from 918 SNPs and row-type phenotype for different association models are presented. The more uniform the distribution of P-values, the better is the model

We tested several models to detect associations between SNP markers and agronomic traits. Owing to the complexity and the considerable amount of population structure present in our panel, we observed numerous spurious associations when using the naive (simple) model for AM. Hence, we assessed the usefulness of various linear models to account for population structure by comparing their ability to reduce the inflation of false positive associations. To this end ranked *P-*values from GWAS were plotted in a cumulative way for each model by using spike morphology as phenotypic trait (Figure 7). As demonstrated by Kang et al. [53] the distribution of *P-*values ideally should follow a uniform distribution with less deviation from the expected *P-*values. The models QK, PK and K showed a good fit for *P-*values, while the other models were characterized by the excess of small *P-*values which is tantamount to an abundance of spurious associations. This is particularly obvious in the case of the "naive" model, where nearly half of the *P*-values are smaller than 0.01. On the other hand, the K-model performed similar to the PK and QK model in displaying a highly uniform distribution of *P-*values and at the same time requiring less computational time. Irrespective of the model, major marker trait associations were constantly detected. However, the more stringent the model was the less spurious background associations were detected. All models considered for GWAS are presented for the trait spike morphology (Additional file 11: Figure S6). For all other traits only results from the K-model will be presented and discussed.

#### Barley spike morphology (row type)

**Figure 8:**
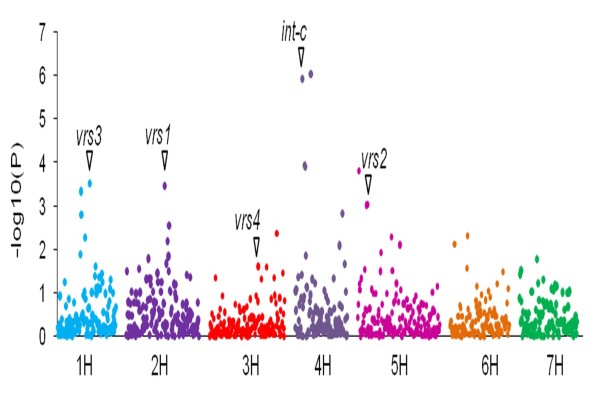
**GWA scan for the trait row type using 918 SNPs with K-model for statistical correction of population structure**. Vertical axis represents -log10(P) values of the *P-*value of the marker trait association. SNPs in the vicinity of the genes *vrs1. vrs2. vrs3, vrs4 *and *int-c *are marked with arrows

Apart from comparing different AM models, we aimed to examine the spike morphology trait as a proof of concept for GWAS and to evaluate the resolution of the association panel. According to its spike morphology barley is classified as two-rowed and six-rowed types and the genes for this trait have been well documented with some of them already cloned [29,30,60]. We scored the row type character in the panel and considered 918 markers for AM using all models. A marker trait association was considered when the marker main effect was significant at 0.03 [-log_10 _(0.03) = 1.5]. This results in a total of 34 markers that are significantly associated with the trait row type by using the K-model. (Additional file 11: Figure S6). The results are congruent with previous row type studies (see Figure 8).

#### Heading date

**Figure 9:**
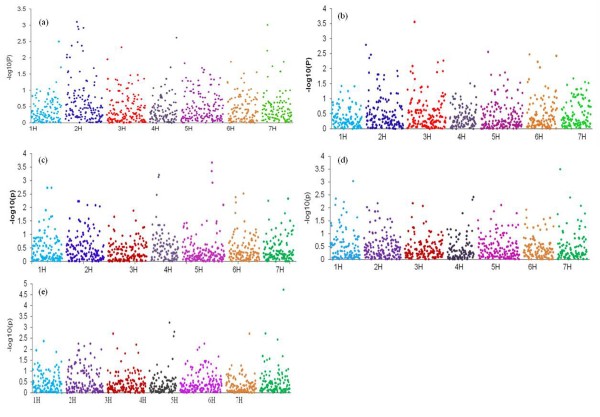
**GWA scans for traits HD (9a), PHT (9b), TGW (9c), SC (9 d) and CPC (9e) using 918 SNPs and the K-model**. Vertical axis represents -log10(P) values of the *P-*value of the marker trait association. The peaks above minimum threshold of 1.5 (*P-*value = 0.03) can be considered as significantly associated

Thirty-four markers were found to be significantly associated with heading date (HD). These were grouped into 19 QTL located on all chromosomes. Significant marker trait associations within a genetic distance of 5-10 cM are delineated into a single QTL. Chromosome 2H harbors the maximum number of markers associated with the trait (Figure 9a). Some of these association results with the SNP markers effectively correspond to genomic regions of previously mapped flowering time QTL. These include genomic regions of various prominent flowering pathway genes like *Ppd-H1, HvFT1, HvCO1 *and *HvCO3 *(see Table 7).

**Table 7:** GWAS results for trait heading date

SNP	Chr	Position	MAF	P-value	-log10(P)	R^2 ^(%)	Effect	QTL	Reference QTL	Literature
SNP111	1H	128.14	0.19	0.0032	2.49	0.63	-2.51	QTL1_HD	*HvFT3*	[61]
SNP119	1H	138.92	0.23	0.0198	1.70	0.33				

SNP129	2H	27.29	0.37	0.0099	2.00	0.38				
SNP130	2H	28.44	0.36	0.0080	2.10	0. 39	-1.29	QTL2_HD	*PpdH1*	[62]
SNP133	2H	31.02	0.38	0.0280	1.55	0.39				[61]
SNP135	2H	33.73	0.10	0.0262	1.58	0.51				

SNP142	2H	41.66	0.27	0.0096	2.02	0.40				
SNP148	2H	53.53	0.34	0.0043	2.37	0.47	-1.37	QTL3_HD		

SNP170	2H	63.53	0.32	0.0007	3.10	0.88				
SNP174	2H	63.53	0.41	0.0011	2.96	0.68				
SNP177	2H	63.53	0.32	0.0013	2.89	0.55		QTL4_HD	*HvFT4/eam6*	[61,63,64]
SNP173	2H	63.53	0.33	0.0033	2.48	0.53				
SNP183	2H	66.83	0.44	0.0265	1.58	0.39	-2.32			

SNP191	2H	71.12	0.44	0.0043	1.0061	0.0012				
SNP196	2H	73.04	0.10	0.0061	2.21	0.65		QTL5_HD	*eps2*	[62]
SNP199	2H	73.75	0.14	0.0012	2.92	0. 49	2.53			

SNP242	2H	115.78	0.39	0.0207	1.68	0. 54	-1.98	QTL6_HD		

SNP284	3H	8.23	0.24	0.0111	1.95	0. 42	-1.45	QTL7_HD		

SNP340	3H	59.89	0.35	0.0047	2.33	0. 38	1.80	QTL8_HD	*HvGI*	[61]

SNP520	4H	82.42	0.32	0.0198	1.70	0.47	-1.15	QTL9_HD		

SNP543	4H	123.29	0.26	0.0024	2.62	0.67	-1.59	QTL10_HD		

SNP559	5H	39.97	0.46	0.0146	1.84	0.32	-1.20	QTL11_HD	*HvCO3*	[65]

SNP630	5H	100.28	0.20	0.0203	1.69	0.58				
SNP635	5H	102.06	0.13	0.0278	1.56	0. 34	-2.02	QTL12_HD		
SNP636	5H	103.92	0.28	0.0278	1.56	0. 35				
SNP639	5H	108.18	0.38	0.0236	1.63	0.53				

SNP728	6H	28.39	0.43	0.0132	1.88	0.28	-1.43	QTL13_HD		

SNP778	6H	60.23	0.49	0.0306	1.51	0. 37	-2.15	QTL14_HD		

SNP829	6H	124.85	0.33	0.0281	1.55	0. 37	1.52	QTL15_HD		

SNP854	7H	37.55	0.36	0.0060	2.22	0.57	2.50	QTL16_HD	*HvFT1*	[61,63]
SNP855	7H	38.32	0.35	0.0009	3.01	0.54				

SNP875	7H	68.46	0.06	0.0180	1.74	0. 48	-2.33	QTL17_HD		

SNP908	7H	84.92	0.35	0.0266	1.58	0. 37	1.66	QTL18_HD	*HvCO1*	[61,65]

SNP921	7H	104.78	0.35	0.0131	1.88	0.59	-1.79	QTL19_HD		

Significant markers associated for heading date with K-model, corresponding MAF, *P *value of association, variance explained by marker (R^2^), effect of the most significant marker within the QTL interval, name of the QTL, and the reference QTL or gene from literature.

#### Plant height

Thirty-two markers displayed significant associations with plant height (PHT). These markers detected 19 QTL (Table 8). Except for chromosome 1H, significantly associated markers were found on all chromosomes with the majority located on 2H and 3H (Figure 9b).

**Table 8:** GWAS results for trait plant height

Marker	Chr	Position	MAF	P-value	-log10(P)	R^2 ^(%)	Effect	QTL	Reference QTL	Literature
SNP122	2H	8.57	0.13	0.0016	2.80	0.94	-5.64	QTL1_PHT		

SNP136	2H	33.74	0.35	0.0138	1.86	0.54				
SNP137	2H	38.03	0.35	0.0044	2.36	0.84	-5.23	QTL2_PHT	*Ph2*	[66]

SNP168	2H	59.21	0.16	0.0229	1.64	0.46				
SNP171	2H	63.53	0.10	0.0155	1.81	0.51	6.73	QTL3_PHT	HT (marker:B15c)	[67]
SNP175	2H	63.53	0.10	0.0155	1.81	0.51				

SNP199	2H	73.75	0.14	0.0224	1.65	0.49	4.72	QTL4_PHT	*sdw3*	[68]
SNP200	2H	74.37	0.25	0.0162	1.79	0.54				

SNP254	2H	130.01	0.20	0.0117	1.93	0.56	-4.57	QTL5_PHT	QHt.StMo-2H.2	[69]
SNP256	2H	131.77	0.28	0.0175	1.76	0.5				

SNP295	3H	36.49	0.13	0.0124	1.91	0.55				
SNP303	3H	43.23	0.23	0.0083	2.08	0.61	-5.57	QTL6_PHT	QHt.HaMo-3H	[67,69]
SNP304	3H	46.31	0.35	0.0141	1.85	0.54				

SNP312	3H	52.50	0.25	0.0002	3.55	1.15				
SNP313	3H	52.50	0.42	0.0220	1.66	0.48	-5.80	QTL7_PHT	*uzu*	Grain genes database

SNP404	3H	126.27	0.39	0.0129	1.89	0.55	4.11	QTL8_PHT	*sdw1/denso*	[70,71]
SNP406	3H	127.10	0.37	0.0061	2.21	0.65				

SNP427	3H	155.09	0.06	0.0120	1.92	0.56	-2.68	QTL9_PHT		
SNP429	3H	162.15	0.06	0.0053	2.28	0.75				

SNP519	4H	80.79	0.18	0.0306	1.51	0.4	3.70	QTL10_PHT	QHei.pil-4H.5	[72]

SNP575	5H	50.27	0.31	0.0028	2.55	0.8	5.30	QTL11_PHT		
SNP588	5H	51.30	0.41	0.0159	1.80	0.5				

SNP623	5H	85.93	0.16	0.0164	1.79	0.51	4.95	QTL12_PHT		

SNP643	5H	110.26	0.10	0.0133	1.88	0.41	-5.58	QTL13_PHT	HT	[71]

SNP654	5H	132.63	0.44	0.0235	1.63	0.46	-4.15	QTL14_PHT	QHei.pil-5H.1	[72]

SNP722	6H	12.54	0.42	0.0229	1.64	0.41	-5.11	QTL15_PHT		
SNP724	6H	16.97	0.30	0.0033	2.48	0.8				

SNP757	6H	55.36	0.09	0.0060	2.24	0.64	-8.54	QTL16_PHT		
SNP766	6H	55.36	0.32	0.0092	2.04	0.6				

SNP831	6H	124.85	0.28	0.0038	2.42	0.74	-4.89	QTL17_PHT		

SNP882	7H	73.75	0.45	0.0210	1.68	0.46	-4.23	QTL18_PHT	HT	[71]

SNP947	7H	144.45	0.41	0.0301	1.52	0.43	-3.77	QTL19_PHT		

Significant markers associated for trait PHT, their MAF, P-value of association, variance explained by marker (R^2^), effect of the most significant marker within the QTL interval, name of the QTL, and the reference QTL or gene from literature.

### Thousand grain weight

Thirty-six markers yielding 21 QTL were significantly associated with Thousand Grain Weight (TGW, Figure 9c). Markers significantly associated with the trait were present on all chromosomes. As expected some of the TGW related QTL overlapped with QTL for spike morphology. The markers SNP56, SNP215, SNP385 and SNP458 are co-localized to the same region as *Vrs3, Vrs1, Vrs4 *and *Int-c *genomic regions (Table 9).

**Table 9:** GWAS results for trait thousand grain weight

Marker	Chr	Position	MAF	P-value	-log10(P)	R^2 ^(%)	Effect	QTL	Reference QTL	Literature
SNP48	1H	55.49	0.47	0.0288	1.56	0.34	-2.19	QTL1_TGW		

SNP56	1H	61.53	0.26	0.0128	1.92	0.39				
SNP62	1H	66.70	0.25	0.0019	2.77	0.70	2.59	QTL2_TGW	*vrs 3*	[60]
SNP68	1H	72.43	0.09	0.0263	1.60	0.37				

SNP76	1H	87.62	0.21	0.0225	1.67	0.38				
SNP78	1H	88.23	0.26	0.0019	2.79	0.69	2.29	QTL3_TGW		
SNP81	1H	92.04	0.28	0.0208	1.70	0.38				

SNP137	2H	38.03	0.35	0.0259	1.61	0.40	-1.20	QTL4_TGW		

SNP171	2H	63.53	0.10	0.006	2.26	0.50				
SNP174	2H	63.53	0.41	0.029	1.55	0.35	2.27	QTL5_TGW	QTgw.pil-2H.2	[67,72]
SNP175	2H	63.53	0.10	0.006	2.26	0.50				

SNP210	2H	82.75	0.36	0.0081	2.13	0.51				
SNP215	2H	86.63	0.32	0.0267	1.59	0.38	1.33	QTL6_TGW	*vrs1*	[60]

SNP245	2H	117.91	0.42	0.0084	2.11	0.51	-1.66	QTL7_TGW		

SNP262	2H	139.65	0.31	0.0091	2.07	0.49	-1.62	QTL8_TGW		

SNP305	3H	47.09	0.16	0.0225	1.67	0.39	3.01	QTL9_TGW		

SNP385	3H	98.49	0.37	0.0131	1.91	0.45	1.94	QTL10_TGW		[60]

SNP395	3H	111.42	0.37	0.0302	1.54	0.36	-1.35	QTL11_TGW	QTgw.S42-2H.a	[73]

SNP458	4H	26.19	0.34	0.0224	1.67	0.40	1.75	QTL12_TGW	*int-c*	[29,60]
SNP460	4H	26.66	0.26	0.0034	2.52	0.63				

SNP467	4H	40.36	0.33	0.0007	3.21	0.74				
SNP469	4H	40.36	0.17	0.0006	3.28	0.82	2.52	QTL13_TGW	QTgw.pil-4H.3	[72]

SNP643	5H	110.26	0.10	0.0312	1.52	0.28	-3.00	QTL14_TGW	QTgw.pil-5H.2	[72]

SNP663	5H	142.2	0.16	0.0004	3.45	0.87				
SNP664	5H	142.2	0.16	0.0002	3.79	1.00	4.47	QTL15_TGW	QGwe.TaER-5H.2	[74]
SNP666	5H	142.2	0.15	0.0012	3.00	0.74				

SNP709	5H	187.38	0.28	0.0082	2.12	0.50	2.02	QTL16_TGW	QTgw.pil-5H.4	[72]

SNP739	6H	43.83	0.08	0.016	1.82	0.43				
SNP740	6H	44.77	0.42	0.0041	2.43	0.6				
SNP741	6H	44.77	0.41	0.0064	2.23	0.55	-1.91	QTL17_TGW		
SNP770	6H	55.94	0.31	0.003	2.58	0.54				

SNP851	7H	34.82	0.43	0.0056	2.29	0.52			QGwe.HaTR-	
SNP854	7H	37.55	0.36	0.0277	1.58	0.32	-1.88	QTL18_TGW	7H.1	[75]

SNP919	7H	88.65	0.13	0.0164	1.81	0.43	3.01	QTL19_TGW		

SNP934	7H	129.91	0.24	0.0048	2.36	0.59	1.84	QTL20_TGW		

SNP944	7H	143.68	0.12	0.0315	1.52	0.27	-1.37	QTL21_TGW	QTw.HaTR-7H.1	[72]

Significant markers associated for TGW, their MAF, P-value of association, variance explained by marker (R^2^), effect of most significant marker within the QTL interval, name of the QTL, and reference QTL or gene from literature.

#### Starch content

Thirty-five markers were found to be significantly associated with the trait Starch Content (SC). These markers formed a total of 25 QTL (Figure 9d). Significantly associated markers for starch content were present on all chromosomes. Similar to TGW markers corresponding to the *Vrs3 *region (SNP56 & SNP66) are significantly associated with starch content. Several significant markers, co-localized with previously mapped genes and QTL for SC (Table 10).

**Table 10:** GWAS results for trait starch content

SNP	Chr	Position	MAF	P-values	-log10(P)	^2 ^(%)	Effect	QTL	Reference QTL	Literature
SNP20	1H	43.28	0.099	0.0076	2.12	0.3	-0.915	QTL1_SC		
SNP22	1H	47.47	0.340	0.0045	2.35	0.34				

SNP36	1H	51.23	0.396	0.0299	1.52	0.2	-0.78	QTL2_SC		
SNP47	1H	55.49	0.495	0.0190	1.72	0.22				

SNP53	1H	60.19	0.309	0.0105	1.98	0.28				
SNP56	1H	61.53	0.264	0.0059	2.23	0.32	1.34	QTL3_SC		
SNP66	1H	69.53	0.474	0.0148	1.83	0.25				

SNP92	1H	101.45	0.288	0.0009	3.04	0.43	-0.70	QTl4_SC		

SNP108	1H	126.01	0.108	0.0236	1.63	0.32	-0.76	QTL5_SC		

SNP136	2H	33.74	0.349	0.0093	2.03	0.28	-0.63	QTL6_SC	Qsch2a	[76]

SNP174	2H	63.53	0.406	0.0142	1.85	0.25				
SNP176	2H	63.53	0.184	0.0315	1.50	0.19				
SNP180	2H	64.24	0.225	0.0066	2.18	0.31	-1.14	QTL7_SC		
SNP181	2H	64.24	0.209	0.0137	1.86	0.26				

SNP192	2H	71.12	0.474	0.0259	1.59	0.21	-0.68	QTl8_SC	QStr.StMo-2H	Grain genes

SNP222	2H	90.10	0.485	0.0277	1.56	0.22	-1.05	QTL9_SC	Qsch2b	[76]

SNP311	3H	51.73	0.214	0.0227	1.64	0.22	-1.15	QTL10_SC		
SNP334	3H	55.57	0.373	0.0067	2.17	0.31				

SNP358	3H	72.26	0.358	0.0087	2.06	0.29	0.96	QTL11_SC		

SNP507	4H	65.05	0.491	0.0160	1.80	0.23	-0.55	QTL12_SC		

SNP539	4H	111.68	0.175	0.0048	2.32	0.33	1.18	QTL13_SC		

SNP543	4H	123.29	0.256	0.0039	2.41	0.35	1.10	QTL14_SC		

SNP599	5H	58.70	0.351	0.0272	1.57	0.21	0.67	QTL_15SC	QStr.StMo-5H	Grain genes
SNP612	5H	65.49	0.445	0.0135	1.87	0.26				

SNP643	5H	110.26	0.104	0.0080	2.10	0.27	-1.79	QTL16_SC		

SNP725	6H	22.35	0.469	0.0117	1.93	0.27	0.73	QTL17_SC		
SNP727	6H	24.36	0.433	0.0244	1.61	0.22				

SNP795	6H	71.08	0.392	0.0282	1.55	0.2	-0.57	QTL18_SC	QStr.StMo-6H	Grain genes

SNP823	6H	112.32	0.299	0.0252	1.60	0.21	-0.81	QTL19_SC		

SNP836	7H	0	0.199	0.0175	1.76	0.24	-0.74	QTL20_SC		

SNP844	7H	12.42	0.096	0.0003	3.50	0.65	-1.72	QTL21_SC	*waxy*	Grain genes

SNP893	7H	78.22	0.127	0.0040	2.40	0.53	0.81	QTL22_SC		

SNP918	7H	87.97	0.297	0.0296	1.53	0.17	0.38	QTL23_SC	Qsch7a	[76]

SNP930	7H	121.09	0.074	0.0083	2.08	0.24	-1.74	QTL24_SC		

SNP951	7H	149.03	0.24	0.0168	1.77	0.27	-0.76	QTL25_SC		

Significant markers associated for SC with K-model, their MAF, P-value of association, variance explained by marker (R^2^), effect of the most significant marker within the QTL interval, name of the QTL, and the reference QTL or gene from literature.

#### Protein content

We found thirty-four markers to be significantly associated with crude protein content (CPC). These markers detected a total of 23 QTL (Figure 9e) and were distributed over all chromosomes. Some of the QTL for protein content overlapped with the QTL regions identified for CPC (Table 11).

**Table 11:** GWAS results for trait crude protein content

SNP	Chr	Position	MAF	*P*-Value	-log10 (P)	R^2 ^(%)	Effect	QTL	Reference QTL	Literature
SNP47	1H	55.49	0.50	0.0044	2.36	0.78	0.74	QTl1_CPC		

SNP97	1H	114.84	0.25	0.0139	1.86	0.56	-0.85	QTL2_CPC		

SNP136	2H	33.74	0.35	0.0310	1.51	0.45	0.39	QTL3_CPC	QPc.nab-2H.1;Qcp2a	[67,76]

SNP170	2H	63.53	0.32	0.0190	1.72	0.54				
SNP173	2H	63.53	0.33	0.0115	1.94	0.62	0.72	QTL4_CPC	QGpc.StMo-2H.2	[67,75]
SNP174	2H	63.53	0.41	0.0055	2.26	0.74				
SNP177	2H	63.53	0.32	0.0116	1.94	0.61				

SNP200	2H	74.37	0.25	0.0071	2.15	0.72	-0.56	QTL5_CPC		
SNP205	2H	78.03	0.40	0.0296	1.53	0.47				

SNP226	2H	96.82	0.23	0.0056	2.25	0.66	-0.90	QTL6_CPC	QPc.nab-2H.1	[67]
SNP227	2H	96.82	0.19	0.0160	1.80	0.55				

SNP244	2H	116.49	0.24	0.0242	1.62	0.48	-0.47	QTL7_CPC	QGpc.HaMo-2H.2	[75]

SNP272	2H	147.94	0.26	0.0103	1.99	0.62	-0.52	QTL8_CPC		

SNP305	3H	47.09	0.16	0.0020	2.70	0.86	-1.466	QTL9_CPC		
SNP322	3H	55.57	0.18	0.0093	2.03	0.64				

SNP357	3H	72.26	0.32	0.0159	1.80	0.51	-0.47	QTL10_CPC		

SNP401	3H	122.14	0.24	0.0062	2.21	0.68	0.60	QTL11_CPC	Qcp3a	[76]
SNP409	3H	130.82	0.41	0.0146	1.84	0.54				

SNP518	4H	79.58	0.45	0.0006	3.22	1.09	-0.75	QTL12_CPC	QGpc.HaTR-4H.2	[77]

SNP531	4H	97.06	0.11	0.0281	1.55	0.45	0.79			
SNP534	4H	101.62	0.16	0.0025	2.60	0.87		QTL13_CPC	QGpc.StMo-4H	[69]
SNP537	4H	108.70	0.21	0.0016	2.80	0.92				

SNP616	5H	74.78	0.51	0.0107	1.97	0.54	-0.814	QTL14_CP	QGpc.HaMo-5H	[67,75]
SNP623	5H	85.93	0.16	0.0082	2.09	0.61				

SNP643	5H	110.26	0.10	0.0055	2.26	0.73	1.52	QTL15_CPC	QGpc.DiMo-5H.2	[78]
SNP699	5H	171.66	0.11	0.0219	1.66	0.53		QTL16_CPC		

SNP807	6H	83.89	0.25	0.0020	2.70	0.91	0.67	QTL17_CPC		

SNP844	7H	12.42	0.10	0.0214	1.67	0.51	0.79	QTL18_CPC		

SNP855	7H	38.32	0.35	0.0019	2.72	0.91	-0.86	QTL19_CPC		
SNP860	7H	46.19	0.26	0.0314	1.50	0.45				

SNP871	7H	61.32	0.24	0.0285	1.55	0.46	-0.50	QTL20_CPC	QPc.nab-7H	[67]

SNP904	7H	80.94	0.22	0.0036	2.44	0.85	-0.68	QTL21_CPC	QGpc.HaTR-7H	[77]

SNP925	7H	112.46	0.37	0.0210	1.68	0.49	0.41	QTL22_CPC		

SNP930	7H	121.09	0.07	0.00001	4.73	1.57	1.54	QTL23_CPC		

Significant markers associated for CPC with K-model, their MAF, P-value of association, variance explained by marker (R^2^), effect of the most significant marker within the QTL interval, name of the QTL, and the reference QTL or gene from literature.

## Discussion

In the present study we describe the application of whole genome association mapping in a panel of diverse spring barley genotypes for agronomic traits. For each of the analyzed traits we identified 19 to 25 QTL. A substantial portion of the derived QTL locations are congruent with previously identified QTL in various biparental mapping populations (Tables 7, 8, 9, 10, 11). GWAS are strongly influenced by the quality of the phenotypic data [79]. In the present study heritabilities for all traits exceeded 0.9 and phenotypic means reflected a broad variation in the panel. The observed differences for two-rowed and six-rowed groups were expected due to their different breeding histories and the pleiotropic effects of spike morphology (Additional file 5: Table S3). Phenotypic variation observed for all traits is higher in the six-rowed group than in the two-rowed group, which is in accordance with the higher genetic diversity of this subgroup (Table 4). A more detailed analysis of population structure revealed six subgroups, which were mostly defined by spike morphology and geographical origin, both of which are known to impinge on the expression of agronomic traits.

### Genetic diversity and population structure

Arguably an association mapping panel should suffice both phenotypic and molecular diversity for the outcome of reliable association results. Owing to the availability of a large number of mapped SNP markers that can be interrogated in a multiparallel manner [26], we were able to achieve a high marker coverage amounting to 1 marker per 1.18 cM. The average PIC (0.30) and Gene diversity (0.33) values observed in this panel of accessions are comparable with the results in previous studies using bi-allelic markers. PIC values differed among chromosomes and among different germplasm subgroups (Tables 3 & 4). Among all chromosomes, the highest average PIC value (0.33) was detected for chromosome 6H-which corresponds to the observations made by Rostoks et al. [24] in a set of European barley cultivars. We determined the population structure in our panel by implementing various approaches (STRUCTURE, PCoA and NJ-dendrogram) and found similar results. Several previous studies e.g. Maliysheva-Otto et al. [57], Rostoks et al. [24], Zhang et al. [58] and Hamblin et al. [80] have shown that growth habit, spike morphology and geographical origin are the major factors that mirror population structure in barley. Since the present study has been restricted to spring barley, spike morphology and geographical origin were the fundamental determinants for population substructuring (G1 to G6) (Figure 3). The 55 landrace accessions included in this panel were distributed among all groups. The subgroups G1, G2 and G3 are mainly six-rowed barleys and the subgroups G4, G5 and G6 include mainly two-rowed barleys. Two-rowed barleys in the panel are more closely related to each other and less diverse than the six-rowed barleys, which is in contrast to the findings of Zhang et al. [58] for Canadian germplasm. While in our panel two-rowed barleys even outnumbered the six-rowed accessions, the reason for their limited diversity might be that the majority originated from Europe. The geographical distribution of the accessions has a major influence on the diversity of alleles sampled in the population [57]. In Europe, two-rowed barley is mainly grown as raw material for malt production. Malting quality is a quantitative trait. The use of a limited number of principal progenitors in the corresponding breeding programs has resulted in the reduction of genetic diversity and in the concomitant formation of a distinct subpopulation as it is seen in our present panel [81].

### LD configuration and consequences

The resolution of LD mapping depends on the extent of LD across the genome and the rate of LD decay with genetic distance [82,83]. Genome-wide LD studies for barley have been previously reported in various populations using different molecular markers such as AFLP, SSR and DArT [57,58,84], with few studies, however, relying on more than 1000 markers. In our panel of spring barley accessions of worldwide origin, intra-chromosomal whole genome LD decays below the critical *r*^2^-value (0.2) within a genetic distance of 5 cM. It needs to be kept in mind that this is an average value, which summarizes substantial intra-chromosomal LD variation. The extent of intra-chromosomal LD for different chromosomes in our panel ranges from 5-10 cM with varying patterns along each chromosome (Additional file 10: Figure S5). Previous studies found various levels of LD decay in different barley populations [9,29,83] and among different chromosomes [24]. The LD decay was more rapid in the study of Comadran et al. [85] probably due to the inclusion of landraces in the collection. Caldwell et al. [9] also showed that LD decays more rapidly in barley landraces compared to elite barley cultivars. Less extensive LD beyond 10 cM has been found in our panel, as the majority of significant LD values above the basal level (33.7%) are due to tightly linked markers. Significant inter-locus LD values of unlinked markers (4%) may be the result of population structure (Table 6). We found some closely linked markers that are in complete Linkage Equilibrium (LE), while some distantly linked markers exhibited high LD values. This reflects the dynamic variation of LD patterns along the chromosomes as it has been shown in this panel at the sequence level for several transcription factors [27]. As to the individual subgroups, the portion of significant *r*^2^-values above the basal level (0.2) is higher within six-rowed than in two-rowed groups indicating high LD in these groups. Interestingly, LD in all subgroups extended beyond 30 cM except for G5 where LD extended to about 20-25 cM (Figure 5). This is most likely because of the larger population size of G5 compared to the other subgroups. The extensive LD observed in the subgroups is probably due to their decreased population size and a concomitant increase in relatedness.

### Genome-wide association mapping

Despite the advantages of GWAS to pinpoint genetic polymorphisms underlying agronomic traits, this approach may suffer from an inflation of false positives due to population structure [4,52,86]. Several statistical models to correct for the effect of population structure have been proposed and tested in previous studies [37,52,87]. Since we detected a considerable amount of structure in the present panel we used linear models to control for population structure and to reduce the false positive associations. Similar to the previous studies of comparing GWAS models in allogamous and autogamous species [37,52], our results suggest that K-model, QK model and PK model performed better than others (Figure 7). Moreover, for the K-model computational time is faster and no additional steps like identifying appropriate population structure (Q-matrix) in the panel are required. Since in an exploratory analysis mostly consistent results were obtained for all three approaches, the K-model was employed in the complete analysis of all traits to avoid redundancy of data. Still it should be kept in mind that correcting for population structure not only reduces the frequency of false positives but also may entail false negatives in situations where a character state is strongly correlated with population structure [28].

In order to confirm the efficiency and resolution of the panel for association mapping using the range of available markers, we re-mapped all 918 SNPs using the K-model. From 918 SNPs, 783 were re-mapped within 10 cM of their original positions. Only 14% of the markers mapped beyond 10 cM. Among the successfully re-mapped markers more than 95% markers are within 5 cM distance from the original map position indicating the mapping resolution of our panel (see Figure 6). Rostoks et al. [24] has used the same approach to evaluate their barley collection for GWAS with a subset of markers and successfully mapped 80% of the markers.

To demonstrate the suitability of the panel and the model for GWAS, we first analyzed spike morphology (row type) (Figure 8). This trait can be easily scored and is important from the agronomic and the domestication point of view. The genetic basis of row type is already well known and several QTL have been mapped and genes have been cloned [29,60,88]. We identified 34 marker-trait associations for this trait (Figure 8). Our identified marker-trait associations for row type are concurrent with all previously identified major loci-*vrs1*[88], *vrs2, vrs3, vrs4 *and *int-c *[29,30]. Additional, less significant associations detected for row type could not be associated to any known major loci, and need to be further explored. These results for row type act as a proof of concept for GWAS in our spring barley panel and reflect the efficiency of GWAS for high resolution QTL mapping in inbreeding species. Some of the row type QTL overlapped with associated regions for other traits, especially with the traits TGW, SC and CPC (Additional file 12: Figure S7). As expected, two-rowed barley has higher TGW than the six-rowed types, as the number of sink organs (kernels) in two-rowed spikes is smaller than in six-rowed spikes. While the effect of spike architecture on TGW is clearly pleiotropic, its influence on SC and CPC is the result of breeding history and end use quality. In case of malting barley, varieties are generally bred for high starch and low protein content. In Europe mostly two-rowed barley is preferred for malting while six-rowed barley is primarily used as feed and is characterized by high protein content [22]. As a result, the two-rowed types in our panel have higher starch content and lower protein content than six-rowed types (Additional file 5: Table S3). As expected, the landraces included in the panel did not show this stratification as they did not underly this selection pressure.

**Figure 10:**
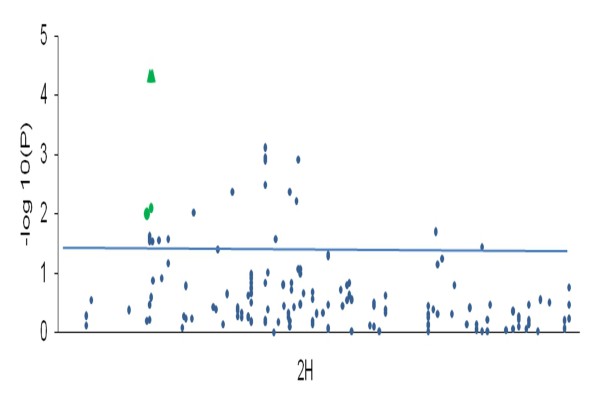
**Association analysis for the trait HD for chromosome 2H with SNPs from IPK-OPA and the resequenced *PpdH1 *fragment **[14]. Blue circles represent the IPK-OPA SNPs on chromosome 2H, Green circles represent the IPK-OPA SNPs that are significantly associated with HD and are in vicinity of the *PpdH1 *gene, green triangles represent SNPs from the re-sequenced *PpdH1 *fragment

Heading date (HD) reflects the adaptation of a plant to its environment and is a complex trait effected by numerous QTL both in outbreeding [89] and in inbreeding species [61]. Many SNP markers were found to be associated with the trait HD (Figure 9a) and we report a total of 34 significant SNPs defining 19 QTL. Some of these QTL hit genomic regions that were previously reported to harbor major genes including *HvFT3, PpdH1, HvFT4, eps2, HvGI, HvCO3, HvFT1 *and *HvCO1 *(Table 7). In a previous study using the same panel, fragments from three flowering time candidate genes were re-sequenced and SNPs within the gene *PpdH1 *revealed the largest effects on HD [14]. In the present GWAS, SNPs located in the vicinity (ca. 2 cM) of *PpdH1 *showed significant associations with HD (Table 7). By further including all *PpdH1 *SNPs from Stracke et al. [14] into our GWAS, these SNPs revealed the highest association of all markers used (Figure 10). These findings lend strength to the hypothesis that a further increase in marker coverage will either lead to the detection of additional associations or improve the significance of existing QTLs.

For the trait PHT we found 19 putative QTL regions located on chromosomes 2H, 3H, 4H, 5H, 6H and 7H comprising 32 marker trait associations. Semi-dwarf and dwarf cultivars have been developed worldwide to reduce lodging and to improve the harvest index. Different genes/alleles have been deployed in different geographic regions: the GA sensitive *sdw1 *dwarfing gene has been deployed in America and Australia, while its allelic form, termed *denso*, is frequently seen in European two-rowed germplasm. The recessive *uzu *allele is found in Japanese, Chinese and Korean cultivars [70,90]. Many QTL for PHT coincide with previously mapped QTL and genes (Table 8). The QTL4_PHT on chromosome 2H coincides with the mapping position of *sdw3 *which plays a major role in gibberellins-insensitive dwarfing barley [68]. Two allelic forms of the dwarfing gene *denso/sdw1 *map to the same genomic region as QTL8_PHT located on the long arm of chromosome 3H [70]. The QTL7_PHT is about 10 cM distant from the *uzu *locus based on the consensus map presented in grain genes database.

Thousand grain weight (TGW) is one of the major yield components having direct effect on the final yield. Altogether 21 QTL were found for this trait and some of them are in vicinity of row type genes. Some of the QTL were further confirmed by previously mapped QTL in same genomic regions (Table 9). QTL14_TGW on 5HL is observed to effect other traits like PHT, SC and CPC.

As outlined above, starch and protein content of the grain are major determinants of the end use quality. Several of the 25 QTL detected for starch content coincided with the previously identified QTL (Table 10). These include QTL for related traits like acid detergent fiber (ADF) content, starch granule size and granule shape [76]. QTL21_SC on 7H is located in the region of the *waxy *locus known to encode *granule-bound starch synthase I *(*GBSS I*), which catalyses the synthesis of amylose [91,92]. For the total grain crude protein content we identified 23 QTL, located on all the seven chromosomes. Eleven of these QTL regions co-localize with previously mapped QTL, while 12 QTL are novel (Table 11). Interestingly, the majority of QTL for traits SC and CPC are located on chromosome 7H. Some of the QTL identified for SC coincide with QTL for CPC e.g. chromosomes 1H (55 cM), 2H (33.74 cM), 3H (55 cM), 5H (110 cM) and 7H (12 cM and 121 cM) (Table 10 & 11). The coincidence of the QTL for these two traits can be expected due to their negative correlation (Table 2). On the other hand, we cannot rule out that some of the shared QTL are the result of linkage of underlying genes.

### GWA reveals small effects only

Even the best associations observed in the present study showed only modest R^2 ^values (percentage of genetic trait variation explained) for the corresponding SNPs, implying low variance predicted by each SNP. This is exemplified by the QTL 'Qsch7a', which in a biparental QTL mapping study explained 47% of variation in SC [76]. In the present study, 'QTL23_SC' located in the same genomic region as 'Qsch7a' explains only 0.2% of the variation. Many GWAS in humans have reported low R^2 ^values and the rest of the unexplained variation is termed as 'unexplained missing heritability' [93]. Roy et al. [94], among others, reported R^2^- values to range from 0.2% to 3.95% in GWAS for plants, which corresponds well with our present results. In a consorted study for the trait "body height", an impressive number of 40 genotypic variants have been identified under a stringent threshold. Together these were only able to explain around 5% of the variation in human body height [95,96]. Possible explanations for this "missing heritability" include i) insufficient marker coverage, in cases where the causal polymorphism is not in perfect LD with the genotyped SNP reduces the power to detect associations and the variation explained by such a SNP marker. This has been demonstrated in the present study for the effect of the *PpdH1 *gene on HD; ii) rare alleles (MAF < 5%) with a major effect have been dropped from the analysis and will go undetected in cases where they are associated; iii) the expression of a character or trait depends on a large number of genes/QTL with small individual effects which escape statistical detection; iv) inadequacy of the statistical approaches available to detect epistatic interactions in GWAS and v) biased estimates of R^2 ^for individual SNPs due to the level of population stratification in the panel [93,95,97-99]. Although the above mentioned reasons were mainly discussed in the context of GWAS in humans, they also pertain to GWAS in plants and other organisms. In addition to the above mentioned reasons, the statistical model employed for the analysis will affect the variation explained by the SNPs. As the stringency and threshold of the models increases, the power of detecting small effect SNPs will be reduced. We observed that in the case of using stringent models for GWAS the larger portion of the trait variation is explained by the model itself and the less variation is left to be explained by genetic effects. For the trait HD the K-model, explained nearly 70% of the variation of the trait. Reducing the stringency of the model would increase the variation explained by the marker, but at the same time would result in more false positives. Especially in inbreeding crops like barley, it is difficult to preclude completely the effect of relationship among genotypes by applying simpler models. Hence, GWAS in highly structured populations of inbreeding crops such as barley will depend on the careful optimization of the model regarding sensitivity vs. selectivity.

## Conclusions

Overall, our results provide new details on the chances and pitfalls of GWAS in structured populations of inbreeding crops like barley. Results from the present study provide an insight into the genetic architecture of important agronomic traits for barley (HD, PHT, TGW, SC and CPC). In total, we identified 107 QTL for these traits. Some genomic regions harbor QTL for more than one trait and, based on map comparisons, 50 QTL have been found to concur with previously mapped QTL. For all traits together, 57 novel QTL have been detected. To mitigate the shortcomings of GWAS in inbreeding crops, future association studies might implement novel strategies such as joint linkage and LD mapping which were already successfully applied in various species [89,100-102]. Furthermore, to fine map and "mendelize" selected QTLs, staggered patterns of LD decay observed for different genepools of barley (cultivars, landraces, wild barley) may be exploited in combination with biparental mapping and marker saturation strategies exploiting the ever increasing body of genomic sequence [30,103]. The feasibility of such an approach was recently demonstrated by identifying a candidate gene for the *ANTHOCYANINLESS 2 *locus using a combination of association mapping followed by a segregation analysis in a biparental population and a BAC contig analysis [104].

## Authors' contributions

RKP carried out the study and performed data curing, association analysis, interpretation of the data and drafted the manuscript. RS participated in statistical analysis and improving the manuscript. MM and FAE advised on statistical analysis. GH generated the phenotypic data. BK coordinated the project, and assisted in GoldenGate data analysis and improving the manuscript. AG initiated the study, participated in data interpretation and in improvement of the manuscript. All the authors have read and approved the final manuscript.

## Supplementary Material

Additional file 1 **Table S1 **Information of 957 mapped SNP markers from the IPK customized OPA that were successful in our panel. Click here for file

Additional file 2 **Table S2 **Details of the 212 accessions used for GWAS. Name of the accession, row type, number of successful markers, Structure group, region of origin and country of origin. Click here for file

Additional file 3 **Figure S1 **STRUCTURE results using DArT markers. Log probability data (LnP(D)) as function of *k *(number of clusters) from the STRUCTURE run using 1088 DArT markers with the same association panel. The plateau of the graph at *K *= 6 indicates the minimum number of subgroups possible in the panel. Click here for file

Additional file 4 **Figure S2 **Phenotypic distribution of 224 spring barley accessions for the traits heading date (HD), plant height (PHT), thousand grain weight (TGW), starch content (SC) and protein content (CPC). Click here for file

Additional file 5 **Table S3 **Phenotypic variation among two-rowed and six-rowed groups. Estimation of means, standard deviation (SD), variation (VAR), standard error variation (SEVAR) and coefficient of variance (CV%) for each trait among two-rowed and six-rowed groups. Click here for file

Additional file 6 **Table S4 **Estimation of means, SD, variation (VAR), standard error variation (SEVAR) and coefficient of variance (CV%) among all six subgroups in the panel. Click here for file

Additional file 7 **Figure S3 **Comparison of BLUPs and BLUEs for starch content. The graph implies that there is not much difference between the BLUPs and BLUEs in our experiment. Click here for file

Additional file 8 **Table S5 **Marker polymorphism information of the 918 SNP markers used in GWAS in the panel. Click here for file

Additional file 9 **Figure S4 **Principal Co-ordinate analysis (PCoA) of the panel based on the first two components derived using 918 SNPs. The primary axis tend to separate into subgroups based on their spike morphology character (blue: six-rowed barley; red: two-rowed barley). Further clustering is based on origin of the accessions. Click here for file

Additional file 10 **Figure S5 **LD plots for each chromosome in barley. The color of squares illustrate the strength of pairwise r^2 ^values on a black and white scale, where black indicates perfect LD (r^2 ^= 1.00) while white indicates perfect equilibrium (r^2 ^= 0). Failed and monomorphic SNPs as well as SNPs with MAF < 0.05 are not considered. Click here for file

Additional file 11 **Figure S6 **GWAS whole genome scans for row type using different association models (naive, P, Q, QK, PK and K). Click here for file

Additional file 12 **Figure S7 **GWAS for all traits. Localization of QTL and candidate genes for the traits row type (RT), heading date (HD), plant height (PHT), thousand grain weight (TGW), starch content (SC) and crude protein content (CPC) on the genetic map with 918 SNP markers. Click here for file
