# Supplementary figures and images for: Genome-wide association studies for agronomical traits in a world wide spring barley collection
Source: BMC Plant Biol. 2012 Jan 27;12:16. doi: 10.1186/1471-2229-12-16 (PMC3349577; doi:10.1186/1471-2229-12-16)

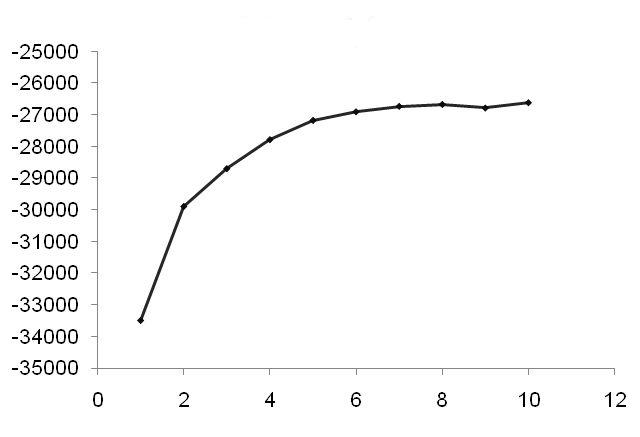

Supplement: Additional file 3 — Figure S1 STRUCTURE results using DArT markers. Log probability data (LnP(D)) as function of k (number of clusters) from the STRUCTURE run using 1088 DArT markers with the same association panel. The plateau of the graph at K = 6 indicates the minimum number of subgroups possible in the panel. [file 1471-2229-12-16-S3.JPEG]

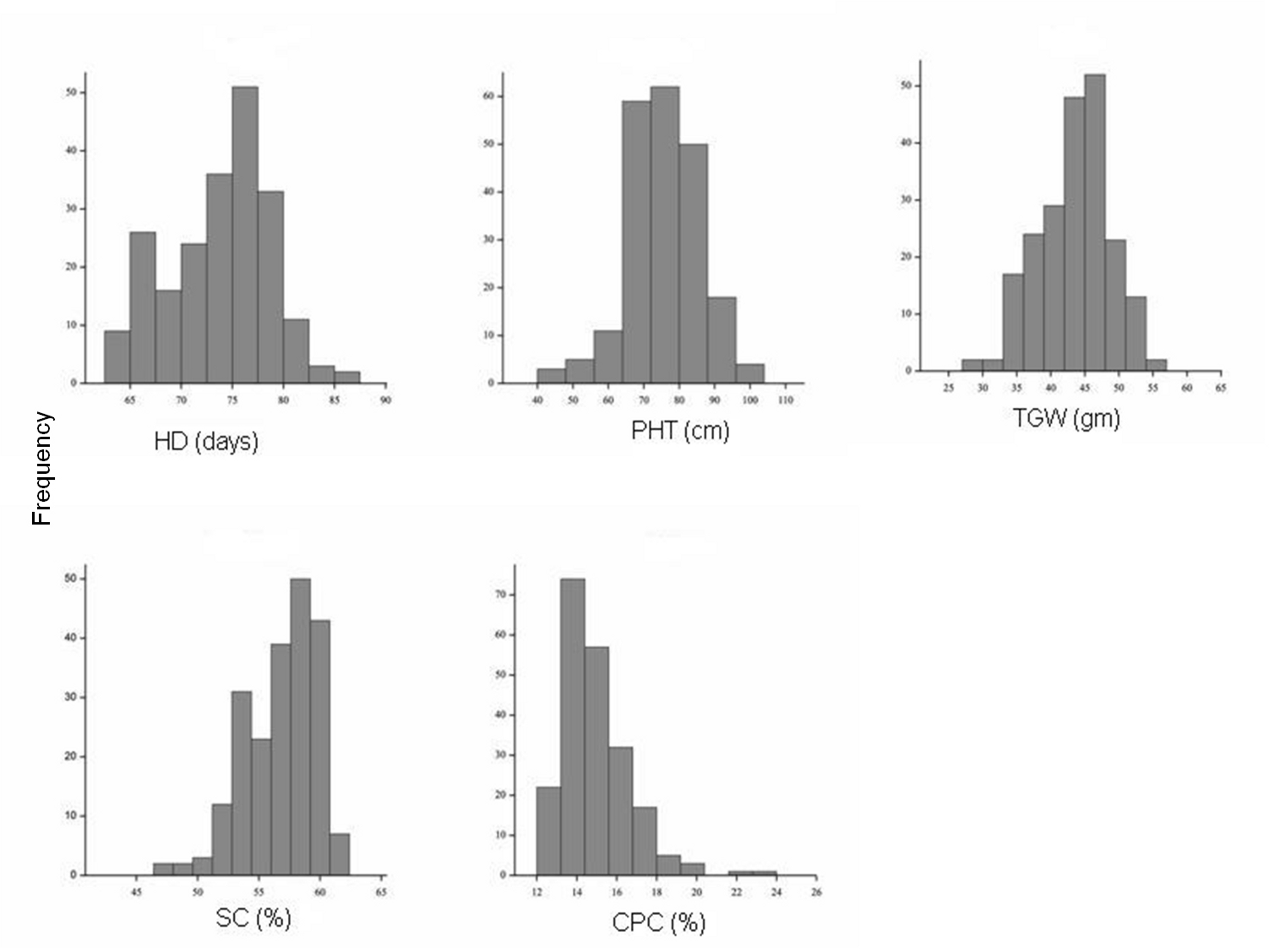

Supplement: Additional file 4 — Figure S2 Phenotypic distribution of 224 spring barley accessions for the traits heading date (HD), plant height (PHT), thousand grain weight (TGW), starch content (SC) and protein content (CPC). [file 1471-2229-12-16-S4.JPEG]

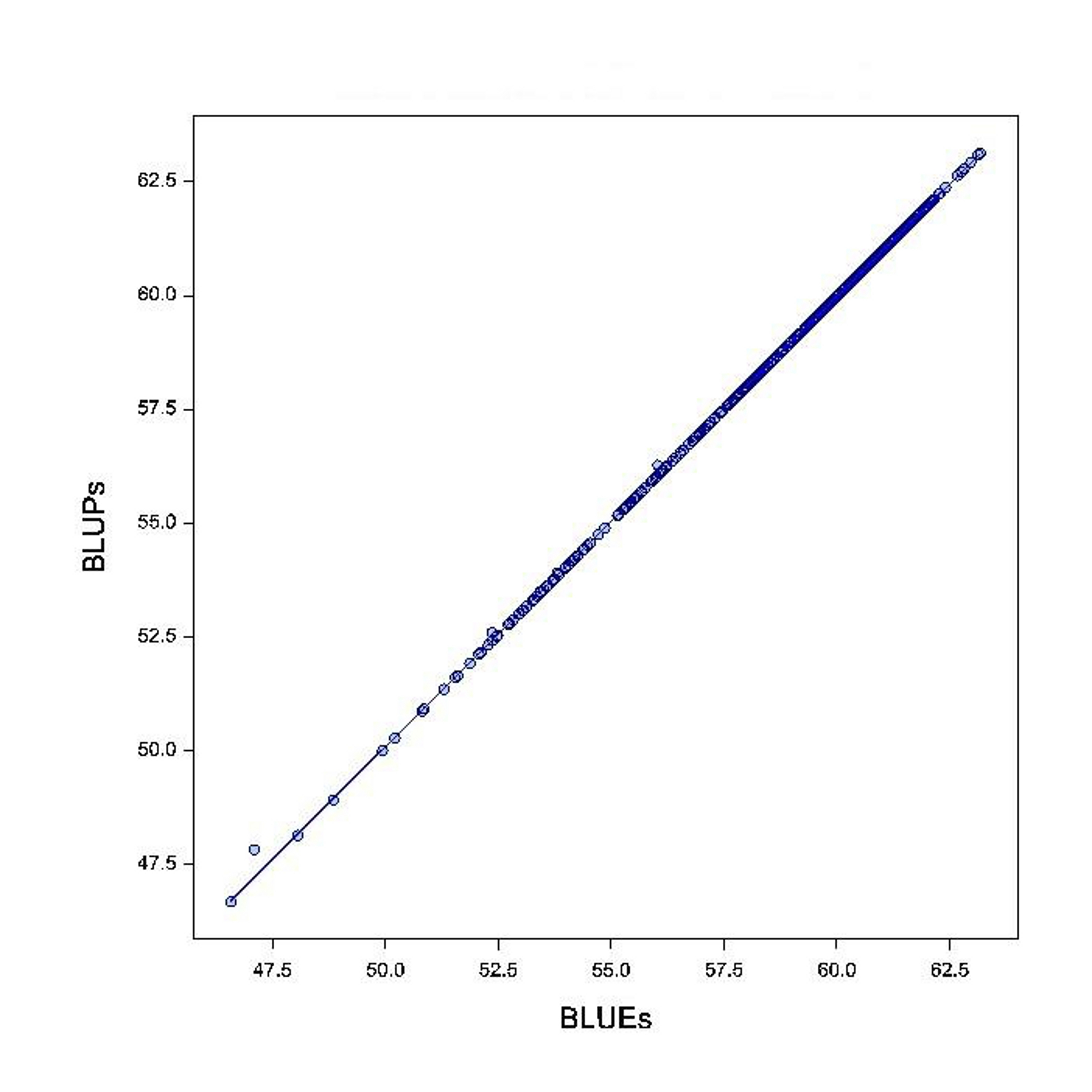

Supplement: Additional file 7 — Figure S3 Comparison of BLUPs and BLUEs for starch content. The graph implies that there is not much difference between the BLUPs and BLUEs in our experiment. [file 1471-2229-12-16-S7.JPEG]

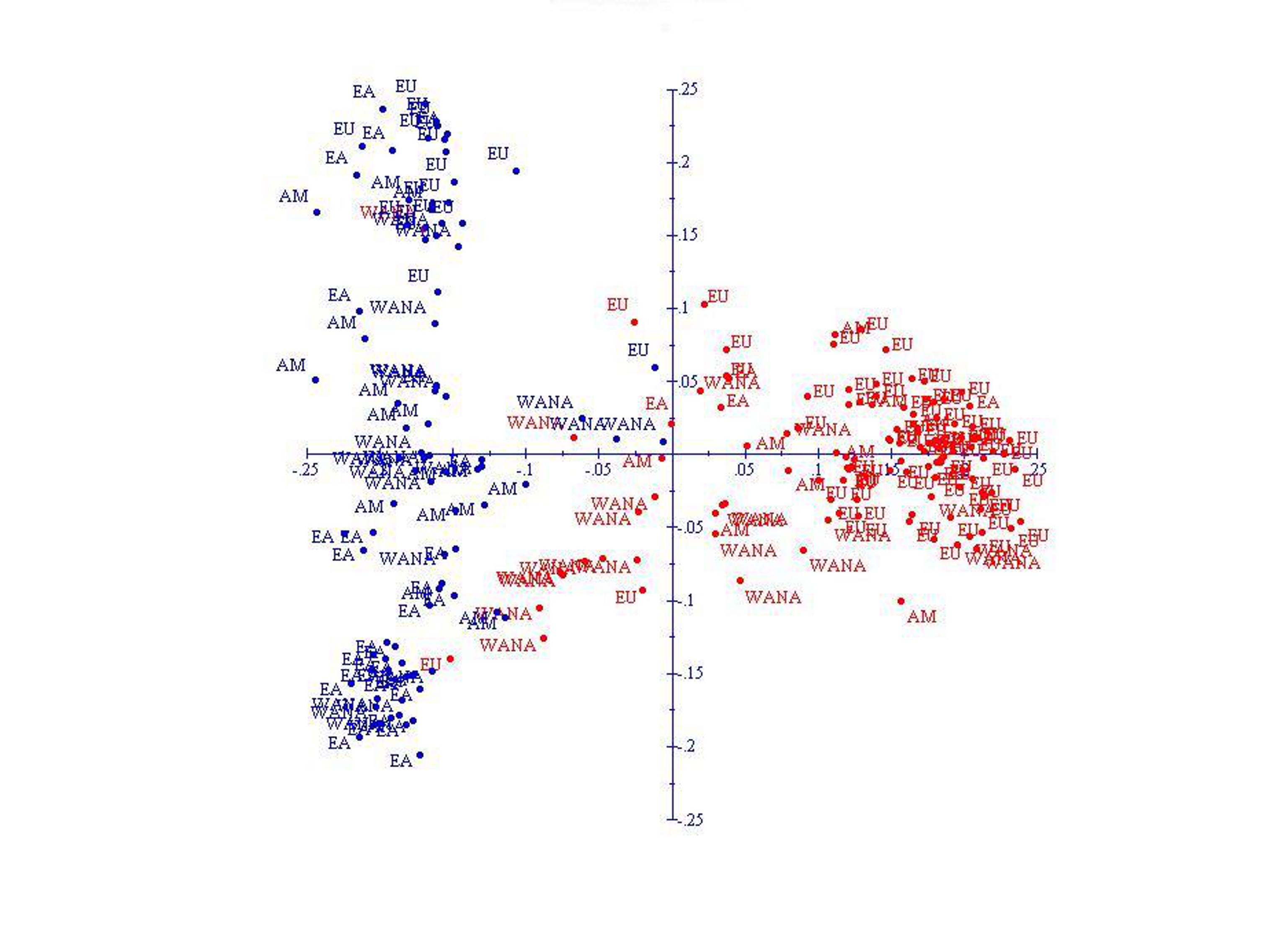

Supplement: Additional file 9 — Figure S4 Principal Co-ordinate analysis (PCoA) of the panel based on the first two components derived using 918 SNPs. The primary axis tend to separate into subgroups based on their spike morphology character (blue: six-rowed barley; red: two-rowed barley). Further clustering is based on origin of the accessions. [file 1471-2229-12-16-S9.JPEG]

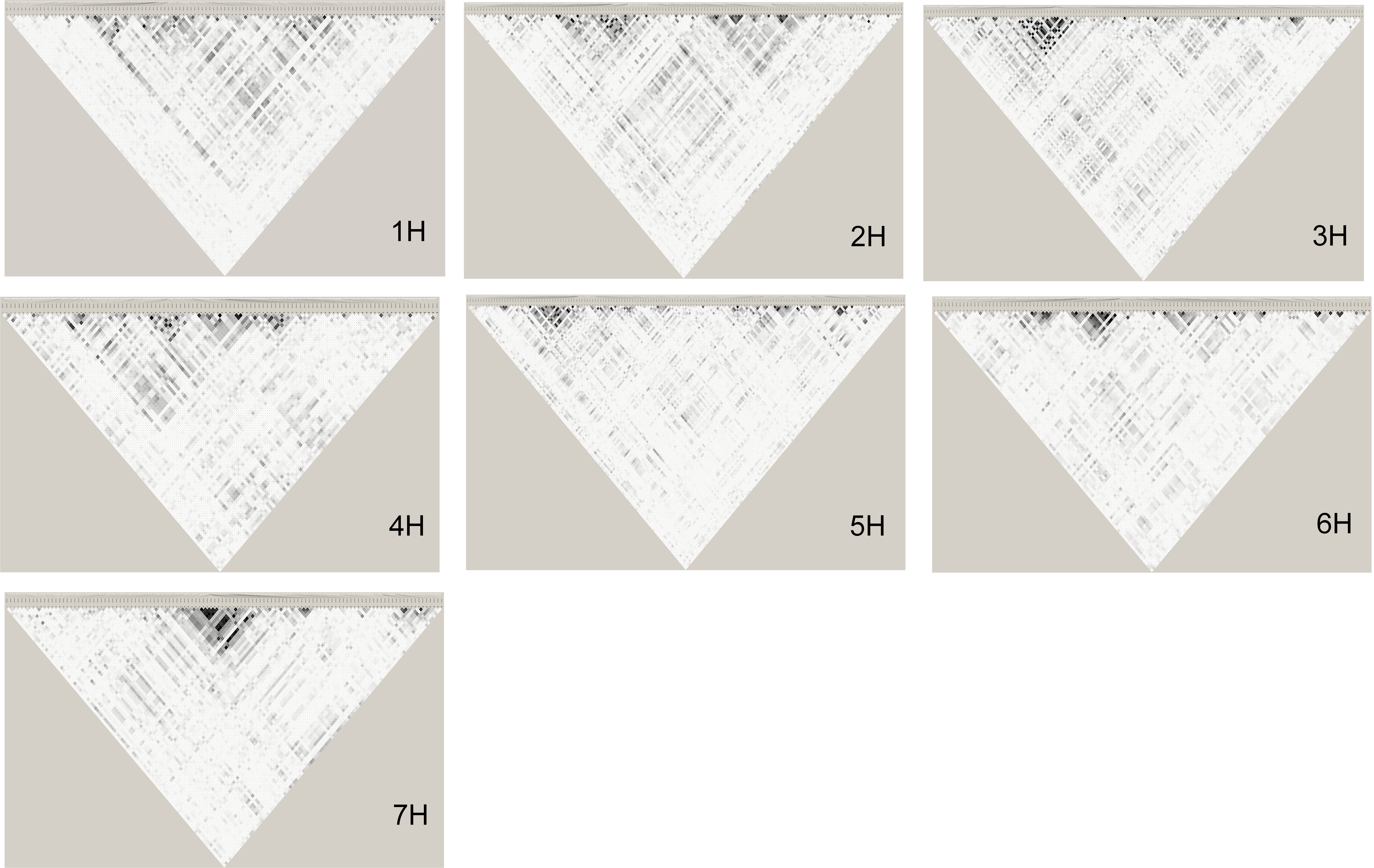

Supplement: Additional file 10 — Figure S5 LD plots for each chromosome in barley. The color of squares illustrate the strength of pairwise r2 values on a black and white scale, where black indicates perfect LD (r2 = 1.00) while white indicates perfect equilibrium (r2 = 0). Failed and monomorphic SNPs as well as SNPs with MAF < 0.05 are not considered. [file 1471-2229-12-16-S10.JPEG]

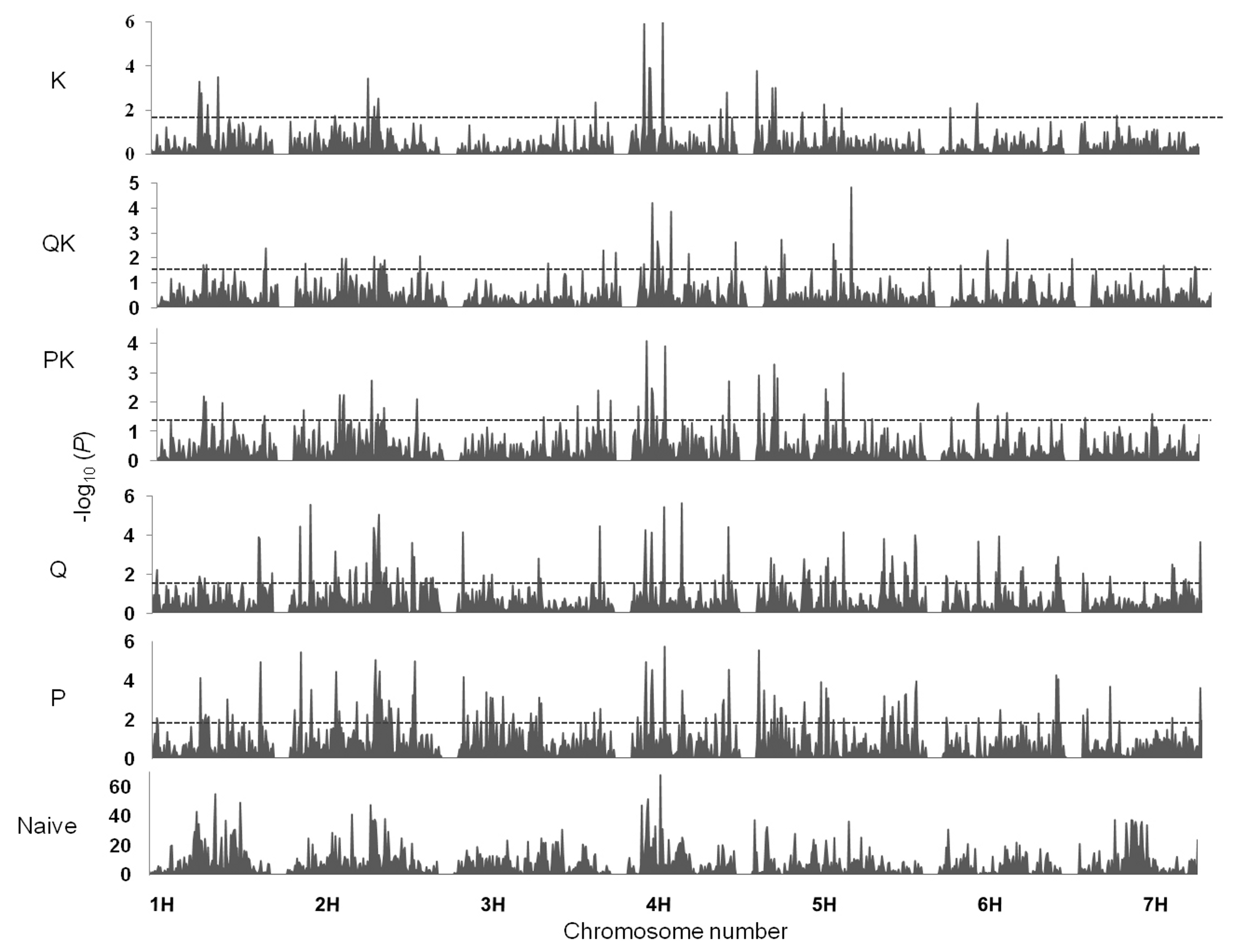

Supplement: Additional file 11 — Figure S6 GWAS whole genome scans for row type using different association models (naive, P, Q, QK, PK and K). [file 1471-2229-12-16-S11.JPEG]

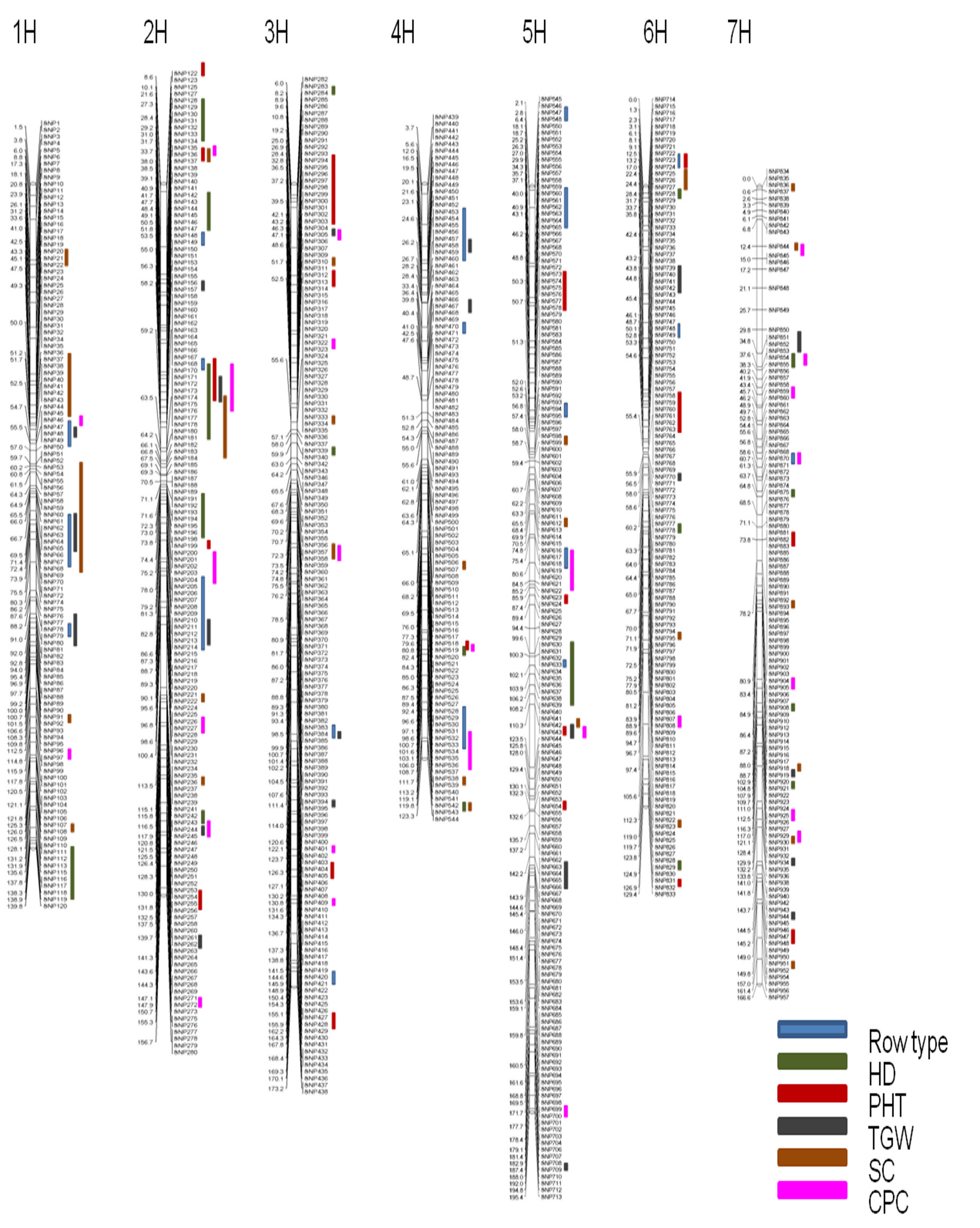

Supplement: Additional file 12 — Figure S7 GWAS for all traits. Localization of QTL and candidate genes for the traits row type (RT), heading date (HD), plant height (PHT), thousand grain weight (TGW), starch content (SC) and crude protein content (CPC) on the genetic map with 918 SNP markers. [file 1471-2229-12-16-S12.JPEG]
