# Supplementary material for: Genome-wide association studies for agronomical traits in a world wide spring barley collection
Source: BMC Plant Biol. 2012 Jan 27;12:16. doi: 10.1186/1471-2229-12-16 (PMC3349577; doi:10.1186/1471-2229-12-16)
Supplement: Additional file 5 — Table S3 Phenotypic variation among two-rowed and six-rowed groups. Estimation of means, standard deviation (SD), variation (VAR), standard error variation (SEVAR) and coefficient of variance (CV%) for each trait among two-rowed and six-rowed groups. [file 1471-2229-12-16-S5.DOCX]

Supplementary Table S3. Estimation of means, standard deviation (SD), variation (VAR), standard error variation (SEVAR) and coefficient of variance (CV%) for each trait among two-rowed and six-rowed groups.

|  | HD | | PHT | | TGW | | SC | | CPC | |
| --- | --- | --- | --- | --- | --- | --- | --- | --- | --- | --- |
|  | 2-rowed | 6-rowed | 2-rowed | 6-rowed | 2-rowed | 6-rowed | 2-rowed | 6-rowed | 2-rowed | 6-rowed |
|  |  |  |  |  |  |  |  |  |  |  |
| MEAN | 75.25 | 71.54 | 75.08 | 75.92 | 46.20 | 40.03 | 58.29 | 54.82 | 14.40 | 15.65 |
| SD | 4.45 | 4.80 | 9.01 | 11.83 | 3.41 | 5.25 | 1.92 | 2.85 | 1.21 | 1.97 |
| VAR | 19.79 | 23.01 | 81.24 | 140.01 | 11.60 | 27.56 | 3.70 | 8.10 | 1.47 | 3.90 |
| SEVAR | 2.76 | 2.82 | 11.91 | 22.79 | 1.44 | 3.82 | 0.51 | 1.29 | 0.25 | 0.87 |
| % CV | 5.91 | 6.71 | 12.01 | 15.59 | 7.37 | 13.11 | 3.30 | 5.19 | 8.44 | 12.61 |
