# Supplementary material for: Genome-wide association studies for agronomical traits in a world wide spring barley collection
Source: BMC Plant Biol. 2012 Jan 27;12:16. doi: 10.1186/1471-2229-12-16 (PMC3349577; doi:10.1186/1471-2229-12-16)
Supplement: Additional file 6 — Table S4 Estimation of means, SD, variation (VAR), standard error variation (SEVAR) and coefficient of variance (CV%) among all six subgroups in the panel. [file 1471-2229-12-16-S6.DOCX]

Supplementary Table S4. Estimation of means, SD, variation (VAR), standard error variation (SEVAR) and coefficient of variance (CV%) among all six subgroups in the panel.

|  |  |  |  |  |  |  |  |
| --- | --- | --- | --- | --- | --- | --- | --- |
|  | Groups | Genotypes | Mean | SD | VAR | SEVAR | % CV |
|  | Total | 212 | 73.68 | 4.94 | 24.40 | 1.93 | 6.70 |
| HD  (days after sowing) | 1 | 23 | 70.86 | 4.69 | 21.95 | 3.65 | 6.61 |
|  | 2 | 31 | 70.42 | 5.16 | 26.60 | 5.85 | 7.32 |
|  | 3 | 31 | 73.57 | 3.95 | 15.60 | 4.57 | 5.37 |
|  | 4 | 24 | 75.41 | 5.22 | 27.24 | 9.08 | 6.92 |
|  | 5 | 79 | 76.13 | 3.42 | 11.69 | 2.79 | 4.49 |
|  | 6 | 23 | 70.83 | 5.18 | 26.88 | 6.98 | 7.32 |
|  | Total | 212 | 75.43 | 10.28 | 105.82 | 11.92 | 13.63 |
| PHT (cm) | 1 | 24 | 73.50 | 7.56 | 57.22 | 19.85 | 10.29 |
|  | 2 | 31 | 69.07 | 9.80 | 96.08 | 31.60 | 14.19 |
|  | 3 | 31 | 85.95 | 8.02 | 64.36 | 15.21 | 9.33 |
|  | 4 | 24 | 83.92 | 9.47 | 89.65 | 36.00 | 11.28 |
|  | 5 | 79 | 74.17 | 7.35 | 54.00 | 13.18 | 9.91 |
|  | 6 | 23 | 67.38 | 9.06 | 82.07 | 22.19 | 13.44 |
|  | Total | 212 | 43.58 | 5.25 | 27.60 | 2.57 | 12.05 |
| TGW (g) | 1 | 24 | 44.05 | 5.78 | 33.45 | 7.77 | 13.13 |
|  | 2 | 31 | 38.10 | 4.15 | 17.21 | 5.15 | 10.89 |
|  | 3 | 31 | 38.89 | 4.79 | 22.91 | 7.00 | 12.31 |
|  | 4 | 24 | 45.19 | 3.87 | 14.95 | 4.41 | 8.56 |
|  | 5 | 79 | 46.07 | 3.08 | 9.48 | 1.42 | 6.68 |
|  | 6 | 23 | 46.54 | 4.24 | 17.96 | 5.10 | 9.11 |
|  | Total | 212 | 56.82 | 2.91 | 8.47 | 0.90 | 5.12 |
| SC (%) | 1 | 24 | 54.64 | 3.04 | 9.27 | 3.19 | 5.57 |
|  | 2 | 31 | 53.64 | 2.84 | 8.07 | 1.82 | 5.30 |
|  | 3 | 31 | 56.52 | 1.82 | 3.30 | 0.79 | 3.21 |
|  | 4 | 24 | 57.18 | 1.70 | 2.89 | 0.96 | 2.97 |
|  | 5 | 79 | 59.19 | 1.32 | 1.75 | 0.38 | 2.23 |
|  | 6 | 23 | 55.26 | 2.11 | 4.47 | 0.82 | 3.83 |
|  | Total | 212 | 14.93 | 1.69 | 2.87 | 0.47 | 11.36 |
| CPC (%) | 1 | 24 | 15.43 | 1.91 | 3.66 | 1.81 | 12.39 |
|  | 2 | 31 | 17.02 | 1.93 | 3.71 | 1.35 | 11.32 |
|  | 3 | 31 | 14.47 | 1.19 | 1.41 | 0.37 | 8.20 |
|  | 4 | 24 | 15.17 | 1.21 | 1.46 | 0.60 | 7.97 |
|  | 5 | 79 | 13.89 | 0.93 | 0.86 | 0.28 | 6.68 |
|  | 6 | 23 | 15.54 | 1.06 | 1.13 | 0.22 | 6.84 |
